# Molecular architecture of language‐related cortical areas revealed by integrative proteomic and connectome analyses

**DOI:** 10.1002/ctm2.70449

**Published:** 2025-08-27

**Authors:** Jinsong Wu, Zixian Wang, Fengjiao Li, Shuolei Bu, Lianglong Sun, Chen Zheng, Zhixin Bai, Luhao Yang, Fangyuan Gong, Jiali Chen, Yien Huang, Wanjing Li, Guoquan Yan, Weiwei Xian, Jiaxuan Yang, Shuai Wu, Kemin Zhu, Wenke Fan, Qiong Liu, Guomin Zhou, Gong‐Hong Wei, Wensheng Li, Jing Yan, Jingliang Cheng, Russell G. Snell, Maurice A. Curtis, Tianye Jia, Binke Yuan, Yong He, Weijiang Zhang, Linya You

**Affiliations:** ^1^ Glioma Surgery Division Neurological Surgery Department of Huashan Hospital Fudan University Shanghai China; ^2^ MOE Key Laboratory of Metabolism and Molecular Medicine and Department of Biochemistry and Molecular Biology of School of Basic Medical Sciences & Fudan University Shanghai Cancer Center Fudan University Shanghai China; ^3^ State Key Laboratory of Common Mechanism Research for Major Diseases Suzhou Institute of Systems Medicine, Chinese Academy of Medical Sciences & Peking Union Medical College Suzhou Jiangsu China; ^4^ Department of Human Anatomy & Histoembryology School of Basic Medical Sciences Fudan University Shanghai China; ^5^ Institutes of Biomedical Sciences Fudan University Shanghai China; ^6^ State Key Laboratory of Cognitive Neuroscience and Learning Beijing Normal University Beijing China; ^7^ Beijing Key Laboratory of Brain Imaging and Connectomics Beijing Normal University Beijing China; ^8^ IDG/McGovern Institute for Brain Research Beijing Normal University Beijing China; ^9^ Institute of Science and Technology for Brain‐Inspired Intelligence Fudan University Shanghai China; ^10^ Key Laboratory of Brain Cognition and Education Sciences Ministry of Education, China: Institute for Brain Research and Rehabilitation, South China Normal University Guangzhou China; ^11^ HanGene Biotech Xiaoshan Innovation Polis Hangzhou China; ^12^ Department of MRI The First Affiliated Hospital of Zhengzhou University Zhengzhou China; ^13^ Applied Translational Genetics Group School of Biological Sciences University of Auckland Auckland New Zealand; ^14^ Centre for Brain Research Auckland University Auckland New Zealand; ^15^ Key Laboratory of Computational Neuroscience and Brain‐Inspired Intelligence (Fudan University) Ministry of Education Shanghai China; ^16^ Centre for Population Neuroscience and Precision Medicine (PONS) Institute of Science and Technology for Brain‐Inspired Intelligence Fudan University Shanghai China; ^17^ Social Genetic and Developmental Psychiatry Centre Institute of Psychiatry Psychology and Neuroscience King's College London London UK; ^18^ School of Psychology University of Southampton Southampton UK; ^19^ Philosophy and Social Science Laboratory of Reading and Development in Children and Adolescents South China Normal University Ministry of Education London China; ^20^ Chinese Institute for Brain Research Beijing China; ^21^ Key Laboratory of Medical Imaging Computing and Computer Assisted Intervention of Shanghai Shanghai China

**Keywords:** bilateral, brain reorganisation, Brodmann area, connectome, human postmortem brain, language, proteomics

## Abstract

**Background:**

Protein expression asymmetry between brain hemispheres is hypothesized to influence functional connectivity, yet its role in language‐related networks remains poorly understood. Additionally, how such molecular differences relate to brain reorganization in glioma requires further exploration.

**Methods:**

We performed label‐free tandem mass spectrometry on 13 left‐hemispheric language‐related Brodmann areas (BAs) and their right‐hemispheric counterparts from 10 donor brains, identifying protein signatures across 6 language‐related functional modules. We then compared these proteomic profiles with resting‐state structural and functional connectivity data from 26 BAs across 90 subjects from the Human Connectome Project (HCP). Finally, we examined functional compensation in 13 glioma patients with tumors in Wernicke's area, correlating gray matter volume in contralateral homologs with linguistic performance.

**Results:**

Protein expression heterogeneity was greater within hemispheres than between homologous contralateral BAs. Hierarchical clustering revealed interactions between core language areas (Broca's, Wernicke's, Geschwind's) and auditory/motor regions. Functional connectivity strength correlated with protein expression similarity, particularly in symmetric BA4 (primary motor cortex). Excitatory/inhibitory (E/I) neuronal markers (GRIA1/GRIA4) showed a left‐positive, right‐negative correlation with connectivity, suggesting hemispheric differences in synaptic regulation. Glioma patients exhibited right‐hemispheric compensation, with gray matter volume in Wernicke's homolog correlating with linguistic function.

**Conclusion:**

Our findings support the hypothesis of a homophilic mixing effect between protein expression similarity and connectome architecture, and help explain brain rearrangement in glioma patients.

**Key points:**

- Protein expression differs more within hemispheres than across homologous regions, with distinct signatures in language‐related brain areas.
- Functional connectivity strength correlates with protein expression similarity, showing left‐right asymmetry in excitatory/inhibitory synaptic regulation (GRIA1/GRIA4).
- Right‐hemispheric homologs compensate for left‐hemispheric language‐area damage in glioma patients, linking molecular profiles to functional reorganization.

## Graphical abstract

Wu et al. studied why certain brain areas are more connected than others. They found that areas with similar protein patterns tend to work together more closely. Additionally, they found when one area is affected such as in brain tumours, other areas with similar patterns especially contralateral regions might help.

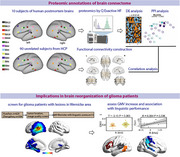

## 1 INTRODUCTION

Annotated connectomes is a new type of theme in neuroscience where brain network structures were superimposed with biological annotations, including gene transcription, neurotransmitter receptors and transporters, cell types and morphology, laminar differentiation, myelination, grey matter morphometry and metabolism.^1^ While Broadmann's early mapping of cortical cytoarchitecture laid a foundation for studying regional heterogeneity,^2^ recent transcriptomic atlases have extended this framework using large‐scale datasets such as BrainSpan,^3^ PsychENCODE^4^ and Allen Brain Institute resources. However, comprehensive protein‐level datasets across the human cortex are still notably limited.

Single‐cell and single‐nucleus transcriptomic atlases have catalogued extensive diversity across brain regions and species. For example, cross‐species profiling of over 450 000 nuclei in primary motor cortex identified conserved excitatory and inhibitory cell types,^5^ while a broader survey of ∼3 million cells revealed 3313 neuronal and glial subtypes aligned with functional specialisations.^6^ The Brain Cell Atlas has further integrated over 26 million cells to standardise annotations and uncover novel microglia and progenitor states.^7^ However, transcript levels only partially reflect functional heterogeneity. Protein abundance is more closely tied to cellular output and can differ significantly from mRNA levels.^8^, ^9^, ^10^ The recently established Brain Protein Atlas addresses this gap using Formalin‐Fixed Paraffin‐Embedded (FFPE)‐compatible proteomics workflows,^11^ but multiregional, bilateral proteomic datasets in the human brain remain scarce.

Language is a uniquely human function supported by a distributed yet asymmetrically organised network, primarily involving frontal, temporal and parietal cortices of the left hemisphere.^12^ While this does not imply that the right hemisphere is completely lacking linguistic ability. In fact, a growing body of evidence indicates that the right cerebral hemisphere also has significant language processing strength.^13^ Core regions such as Broca's area (Brodmann area [BA]44/45) and Wernicke's area (posterior BA22/21) are crucial for syntactic and semantic processing.^14^, ^15^, ^16^ Adjacent areas—including primary auditory cortex (pAC, BA41/42), fusiform gyrus (BA37), Geschwind's territory (BA39/40), ventral premotor cortex (vPMC, BA6/4), ventral sensorimotor cortex (vSMC, BA4 and BA3/1/2) and middle frontal gyrus (BA9)—participate in various aspects of phonological, articulatory, semantic and integrative functions.^17^, ^18^, ^19^, ^20^, ^21^, ^22^, ^23^ These regions are features in dual stream^24^ and oscillation‐based model of language.^25^

Although traditionally viewed as left‐lateralised, recent evidence underscores the right hemisphere's role in language processing and recovery following injury.^26^ For example, glioma patients often exhibit recruitment of right‐hemispheric homologues, particularly when left‐lateralised areas are compromised.^26^ Such compensation may be shaped not only by large‐scale connectivity but also by shared molecular features between homologous regions.

Previous studies have shown that spatial variations in functional connectivity (FC) and structural connectivity (SC) correlate with gene expression profiles.^27^, ^28^, ^29^ Genetic programs involved in synaptic signalling, ion channels and neurodevelopment have been linked to connectome topology and disease vulnerability.^30^, ^31^ However, transcriptomic data offer limited spatial coverage and may not fully capture post‐transcriptional modifications. Protein expression—particularly of synaptic and signalling molecules—is therefore a critical yet underexplored dimension for understanding brain network organisation.

In this study, we conducted a bilateral proteomic analysis of 13 language‐related BAs from 10 postmortem human brains using label‐free tandem mass spectrometry. These regions—spanning Broca's and Wernicke's areas, auditory cortex, fusiform gyrus, Geschwind's territory, sensorimotor cortex and middle frontal gyrus—were analysed alongside resting‐state FC and SC data from 90 Human Connectome Project (HCP) participants. We hypothesised that regions with similar protein profiles, particularly contralateral homologues, would exhibit stronger connectivity. We also examined excitatory/inhibitory (E/I) protein expression balance, and explored functional compensation in 13 glioma patients with Wernicke's area lesions screened out from 1124 glioma patients.

## 2 RESULTS

### 2.1 Multimodal dataset of human language‐related brain regions in both hemispheres

**FIGURE 1:**
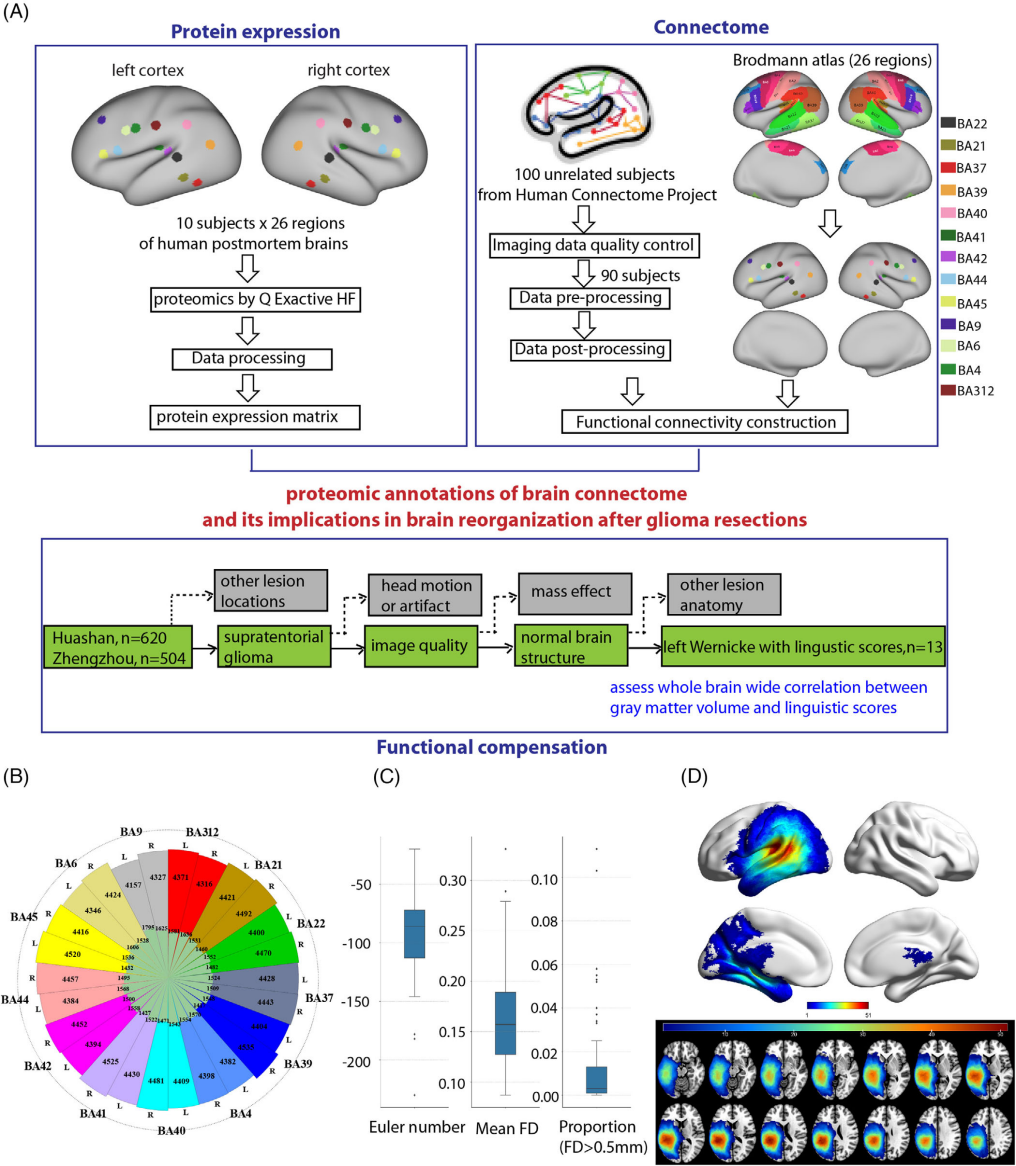
Workflow and overall data quality of protein expression, connectome and functional compensation. (A) Thirteen cortical Brodmann areas (BAs) (BA44, BA45, BA22, BA21, BA39, BA40, BA41, BA42, BA6, BA4, BA3/1/2, BA37 and BA9) of both hemispheres from 10 human postmortem brains were collected for proteomic analyses. Using quality‐controlled MRI images of 90 unrelated subjects from the Human Connectome Project (HCP), a group‐level functional connectivity matrix of 26 BAs in HCP 32k_fs_LR surface space was constructed. Thirteen glioma patients in the Wernicke's area and at the same time had linguistic scores were screened out from 1124 glioma patients. (B) The quantified proteins across multiple BAs of both hemispheres. The outermost ring represented sample types. The second ring (in rainbow colour) referred to the proteins used for differential expression analysis in each BA. The third ring (in light green) represented the proteins excluded in each BA due to low label‐free quantification (LFQ) value. (C) Imaging data quality control indices for each subject in HCP dataset. The figure sequentially displays the Euler number for quantifying surface reconstruction of structural images, the mean frames displacement (FD) and the proportion of frames exhibiting FD greater than .5 mm for quantify head motion during resting‐state fMRI scanning. (D) Lesion‐frequency maps of gliomas in Wernicke's area. Fifty‐two patients with gliomas involving the Wernicke's area were screened from 1124 glioma patients. For each patient, the tumour territory was manually drawn slice by slice on the native three‐dimensional T1‐weighted images and then spatially normalised into Montreal Neurological Institute space. The colour bar represents the number of patients with a lesion at a specific voxel.

For proteomic dataset, we collected 13 language‐related BAs bilaterally from 10 human postmortem brains, including Wernicke's area (BA22/21), Broca's area (BA44/45), Geschwind area (BA39/40), vPMC (BA6/4), vSMC (BA4/3‐1‐2), pAC (BA41/42), BA37 and BA9 (Figure 1A and Table S1). Protein were extracted, digested and analysed via label‐free tandem mass spectrometry (Q Exactive HF, Thermo). On average, ∼5950 proteins were detected per region; after filtering, 4415 high‐confidence proteins were used for analysis (Figure 1B).

For connectome dataset, we mapped the same 26 BAs to cortical surfaces (5 mm radius Region of Interest [ROIs]) in the 32k_fs_LR space according to the anatomical position in postmortem tissue and whole‐brain Brodmann atlas^2^, ^32^ (Figure 1A), and we constructed the group‐level resting‐state FC of the 26 BAs from 90 unrelated HCP subjects.^33^ All data passed HCP standard and additional in‐house quality checks for segmentation, registration and head motion (Figure 1C).

For glioma dataset, from 1124 glioma patients screened from Fudan University affiliated Huashan Hospital and Zhengzhou University affiliated First Hospital, 52 patients with Wernicke's area involvement passed quality control; 13 of these completed linguistic tests (Figure 1A and Table S2). Tumours were manually traced and normalised into Montreal Neurological Institute space (Figure 1D). Grey matter volumes (GMVs) and language scores were compared with 30 healthy controls (HCs) using ROI‐based morphometry (VBM) and correlation analyses.

### 2.2 Assessment of data quality and inter‐individual variability

To assess inter‐donor reproducibility, we calculated the coefficient of variation for each protein across the 10 brains within each of the 13 BAs, summarising these as a median value (V_inter) per protein using normalised Label‐Free Quantification (LFQ) intensities. Nearly half of the proteins exhibited V_inter values below .05 (Figure S1A), indicating high consistency across individuals and supporting the technical robustness of the dataset.

To further validate the dataset, we compared protein expression variability with independent positron emission tomography (PET) and autoradiography data for three receptors with well‐established in vivo profiles. The GABA‐A α1 subunit GABRA1 and the metabotropic glutamate receptor GRM5, located at the low‐variability end of our distribution, show high test‒retest reliability in PET studies (intraclass correlation coefficient [ICC] > .8; variability ≤ 12%)^34^, ^35^ (Figure S1A). By contrast, the cannabinoid receptor CNR1, positioned at the high‐variability extreme, exhibits a between‐subject PET variability of ∼52% and test‒retest variability of ∼15%.^36^ Donor‐by‐region heatmaps for these three receptors (Figure S1B‒D) visually reinforce this contrast.

We also applied differential stability (DS) scores^37^ to evaluate protein consistency across individuals. The DS distribution was centred around 0 (median .03), indicating modest variation overall (Figure S2A). Seven highly stable proteins (Δ*S* > .70) include axonal adhesion proteins (CNTN6, ASTN2), synaptic kinase (PRKCG) and several mitochondrial enzymes, suggesting conserved neuronal and metabolic functions (Figure S2B). In contrast, 31 proteins exhibited low stability (Δ*S* < ‒.30) such as NRAS, calcium channel auxiliary subunit CACNG7, and immune‐related proteins such as IGSF3, reflecting either biological heterogeneity or variable detectability (Figure S2C).

We also compared regional mRNA and protein abundance across the 13 BAs for 4865 shared genes (see Section 4 for the spin test) (Figure S3A,B).^38^, ^39^ Only 59 (1.2%) were classified as Concordant (*ρ* ≥ .50 and false discovery rate (FDR) < .05 after spatial correction) (e.g., GSTT1; *ρ* = .91), and these genes were enriched for redox, mitochondrial and stress‐response functions,^40^ while 255 (5.2%) were Discordant (*ρ* ≤ −.20 and FDR < .05) (e.g., mitochondrial‐fission factor; *ρ* = ‒.57, involved in vesicle trafficking and translation^41^) (Figure S3D). The great majority (∼93.6 %) showed no significant spatial coupling (Figure S3C), consistent with previous large‐scale studies in the adult human cortex,^42^, ^43^ and highlighting widespread post‐transcriptional regulation in the human brain.

### 2.3 Distinct regional and module‐specific protein expression signatures

**FIGURE 2:**
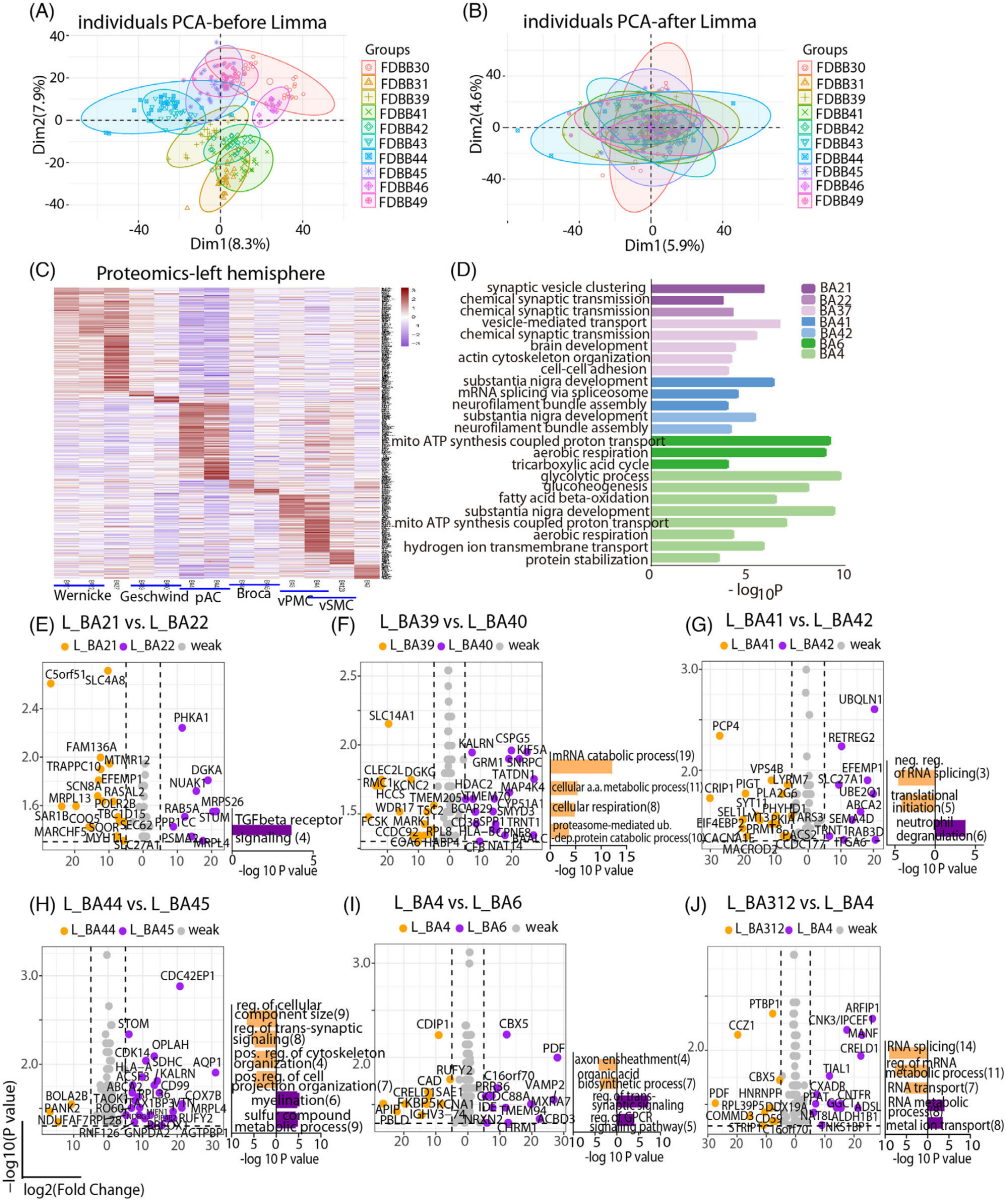
Distinct protein expression patterns in the left language‐related functional modules. (A and B) Principal component analysis (PCA) plots showed the distribution of protein profile of each Brodmann area (BA) from each donor before and after removing batch effect using Limma (A and B, respectively). (C) Heatmap indicated the protein signatures of the left 13 BAs from 10 donors. Functional modules Wernicke, Geschwind, primary auditory cortex (pAC), Broca, ventral premotor cortex (vPMC) and ventral sensorimotor cortex (vSMC) showed distinct protein expression patterns. (D) Selected gene ontology‐biological process (GO‐BP) functional enrichment of differentially expressed proteins (DEPs) of each left BA was shown. BA39, BA40, BA44, BA45, BA312 and BA9 showed no enrichment. False discovery rate (FDR) < .05 and gene count > 2. (E‒J) Pair‐wise differential expression and GO‐BP enrichment analysis between left BA21 and BA22, BA39 and BA40, BA41 and BA42, BA44 and BA45, BA4 and BA6, BA312 and BA4, respectively. DEPs were set with *p* < .05 and avg_log_2_FC > .1. For those DEPs with avg_log_2_FC ≥ 5, they were indicated as orange or purple dots.

To ensure data quality, proteins not detected in at least five of 10 donors per BA were excluded. Principal component analysis initially revealed clustering by individual (Figure 2A); applying the Limma algorithm^44^ corrected batch effects and improved cross‐sample distribution (Figure 2B). In the left hemisphere, six language‐related functional modules (Wernicke's, Broca's, Geschwind's, vPMC, vSMC and pAC) showed distinct protein expression profiles (Figure 2C and Table S3). Gene ontology‐biological process (GO‐BP) enrichment analysis revealed functional specialisation: Wernicke's area was enriched in synaptic transmission, pAC in neurofilament and substantia nigra development, and vPMC and BA4 in metabolic and mitochondrial processes (Figure 2D and Table S4, FDR < .05). No significant GO‐BP terms were enriched in Geschwind's or Broca's areas.

Differential expression analysis within each module identified region‐specific proteins (differentially expressed proteins [DEPs] with *p* < .05, log_2_FC ≥ 5 were coloured; Table S5). In Wernicke's area, BA22 showed high NUAK1 expression, a critical regulator of TGFβ signalling,^45^ consistent with enrichment in TGFβ receptor signalling (Figure 2E and Table S6, FDR < .05). BA39 of Geschwind's area expressed metabolic proteins such as FCSK, HCCS and TSC2, with GO terms linked to respiration and protein catabolism (Figure 2F). BA41 (pAC) was enriched for EIF4EBP2 and translational initiation (Figure 2G). BA45 (Broca's area), showed higher levels of KALRN,^46^ RBFOX1 (a FOXP2 direct target)^47^ and CDK14,^48^ with enrichment in myelination‐related pathways (Figure 2H). In BA6 (vPMC), synaptic proteins VAMP2^49^ and NRXN2^50^ were upregulated, linked to trans‐synaptic signalling (Figure 2I). Within vSMC, BA312 was enriched in PTBP1 and RNA splicing,^47^ while BA4 expressed MANF^48^ and ADSL,^51^ associated with RNA metabolism (Figure 2J).

Similar region‐specific signatures and enriched pathways were observed in the right hemisphere (Figure S4 and Tables S7‒S10). To quantify inter‐regional similarity, we computed 13 × 13 Spearman correlation matrices of average protein profiles per BA. Although correlations are uniformly high (.97‒1), hemisphere‐specific clustering patterns revealed subtle lateralised differences of language modules at the proteomic level (Figure S5).

In summary, language‐related cortical modules display distinct protein expression patterns and functional specialisations, with consistent findings across hemispheres. These differences span synaptic signalling, metabolism, myelination and RNA processing.

### 2.4 Protein interaction networks reveal region‐specific functional clusters

**FIGURE 3:**
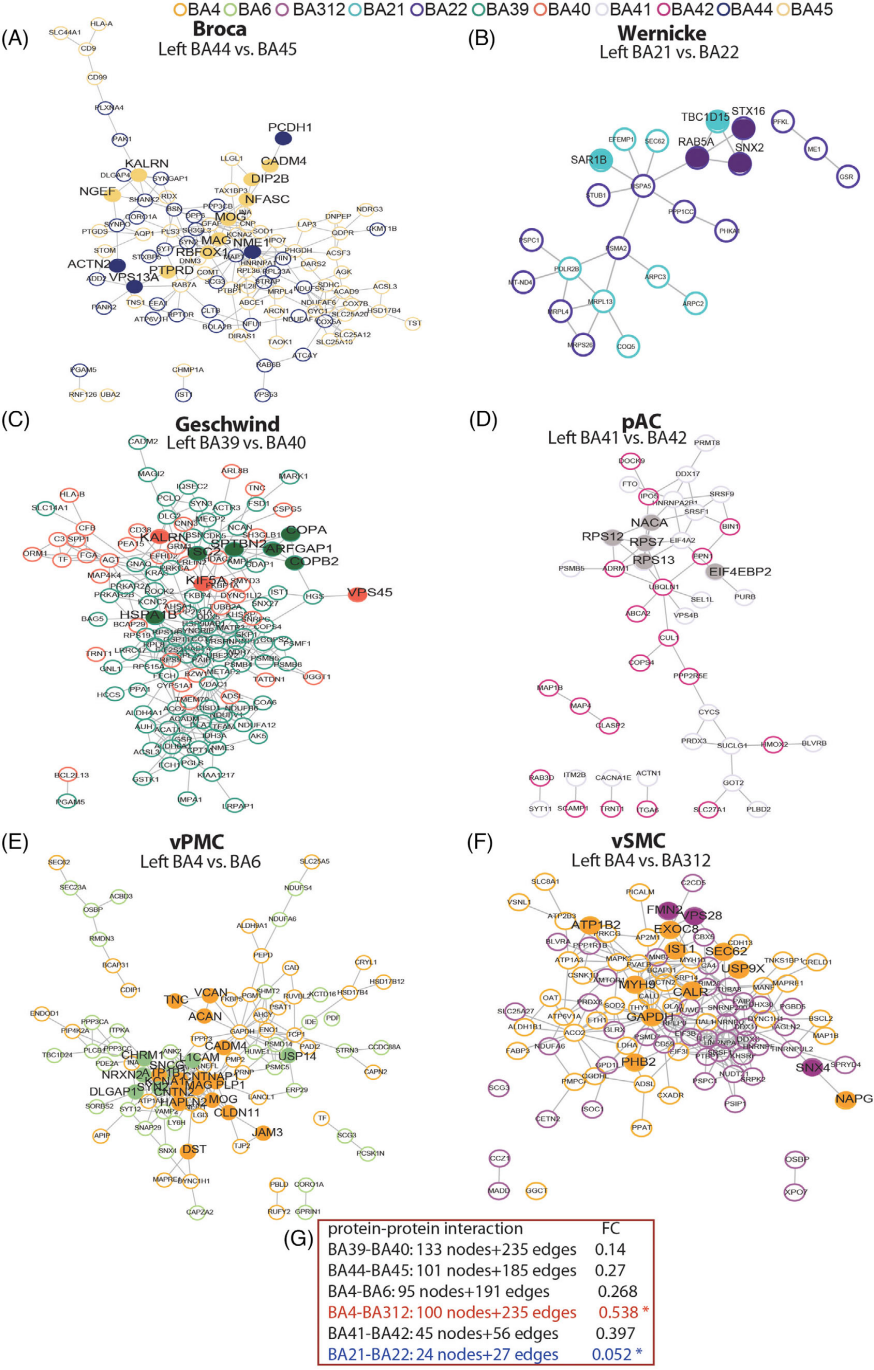
STRING protein–protein interaction (PPI) analysis of the differentially expressed proteins (DEPs) within each language module in the left hemisphere. STRING PPI analysis was performed to evaluate the connectivity of the DEPs between each two Brodmann areas (BAs) of Broca, Wernicke, Geschwind, primary auditory cortex (pAC), ventral premotor cortex (vPMC) and ventral sensorimotor cortex (vSMC) area, respectively, in the left hemisphere. (A) STRING PPI analysis was conducted on DEPs between BA44 and BA45 within Broca's area in the left hemisphere. The network contains 101 nodes with 185 edges. The hollow blue and yellow nodes represent DEPs from BA44 and BA45, respectively. The full coloured nodes denote gene ontology (GO) term ‘nervous system development’ and ‘cell adhesion’. (B) Similar analysis was conducted on DEPs between BA21 and BA22 within the left Wernicke's area. The network contains 24 nodes with 27 edges. The hollow cyan and royal purple nodes represent DEPs from BA21 and BA22, respectively. The full coloured nodes denote ‘intracellular protein transport’. (C) For Geschwind's area, the network contains 133 nodes with 235 edges. The hollow green and red nodes represent DEPs from BA39 and BA40, respectively. The full coloured nodes denote ‘vesicle‐mediated transport’. (D) For pAC area, the network contains 45 nodes with 56 edges. The hollow grey and violet red nodes represent DEPs from BA41 and BA42, respectively. The full coloured nodes denote ‘translation’. (E) Within vPMC area, the network of BA4 and BA6 contains 95 nodes with 191 edges. The hollow orange and light green nodes represent DEPs from BA4 and BA6, respectively. The full coloured nodes form ‘cell adhesion’ and ‘chemical synaptic transmission’. (F) Within vSMC area, network of BA4 and BA312 forms 100 nodes with 235 edges. The hollow orange and purple nodes represent DEPs from BA4 and BA312, respectively. The full coloured nodes form ‘protein transport’ and ‘protein stabilisation’. (G) The comparison of PPI and functional connectivity strength within each module in the left hemisphere.

To identify core pathways within each language‐related module, we performed protein–protein interaction (PPI) analysis of DEPs using the STRING database.^52^ In the left hemisphere, Broca's area showed two densely connected clusters related to nervous system development (e.g., NME1, RBFOX1, KALRN) and cell adhesion (e.g., MAG, MOG, PCDH1) (Figure 3A, full coloured nodes). Wernicke's area was dominated by intracellular protein transport (e.g., RAB5A, STX16) (Figure 3B). Geschwind's area featured vesicle‐mediated transport (e.g., COPA, TSC2, VPS45) (Figure 3C). pAC area showed enrichment in translation‐related proteins (e.g., EIF4EBP2, RPS13) (Figure 3D). Within vPMC area, BA4 and BA6 exhibited clusters in cell adhesion (e.g., L1CAM, CADM4) and chemical synaptic transmission (e.g., CHRM1, NRXN2) (Figure 3E). Similarly, vSMC area showed modules linked to protein transport (e.g., VPS28) and stabilisation (e.g., CALR, USP9X) (Figure 3F).

Overall, Broca's (101 nodes/185 edges), Geschwind's (133/235), vPMC (95/191) and vSMC (100/235) areas exhibited more extensive PPI interactions compared to Wernicke's (24/27) and pAC (45/56) areas, consistent with the observed FC within these modules (Figure 3G).

In the right hemisphere, analogous modules formed strong clusters related to protein transport, mitochondrial respiration, vesicle‐mediated transport and ion transport (Figure S6). Notably, the right vSMC area (91/195) showed the highest network density and strongest FC, while the right Wernicke's area (64/89) had the weakest—mirroring the left‐hemispheric pattern (Figure S6).

### 2.5 vSMC area demonstrated the strongest protein expression asymmetry

**FIGURE 4:**
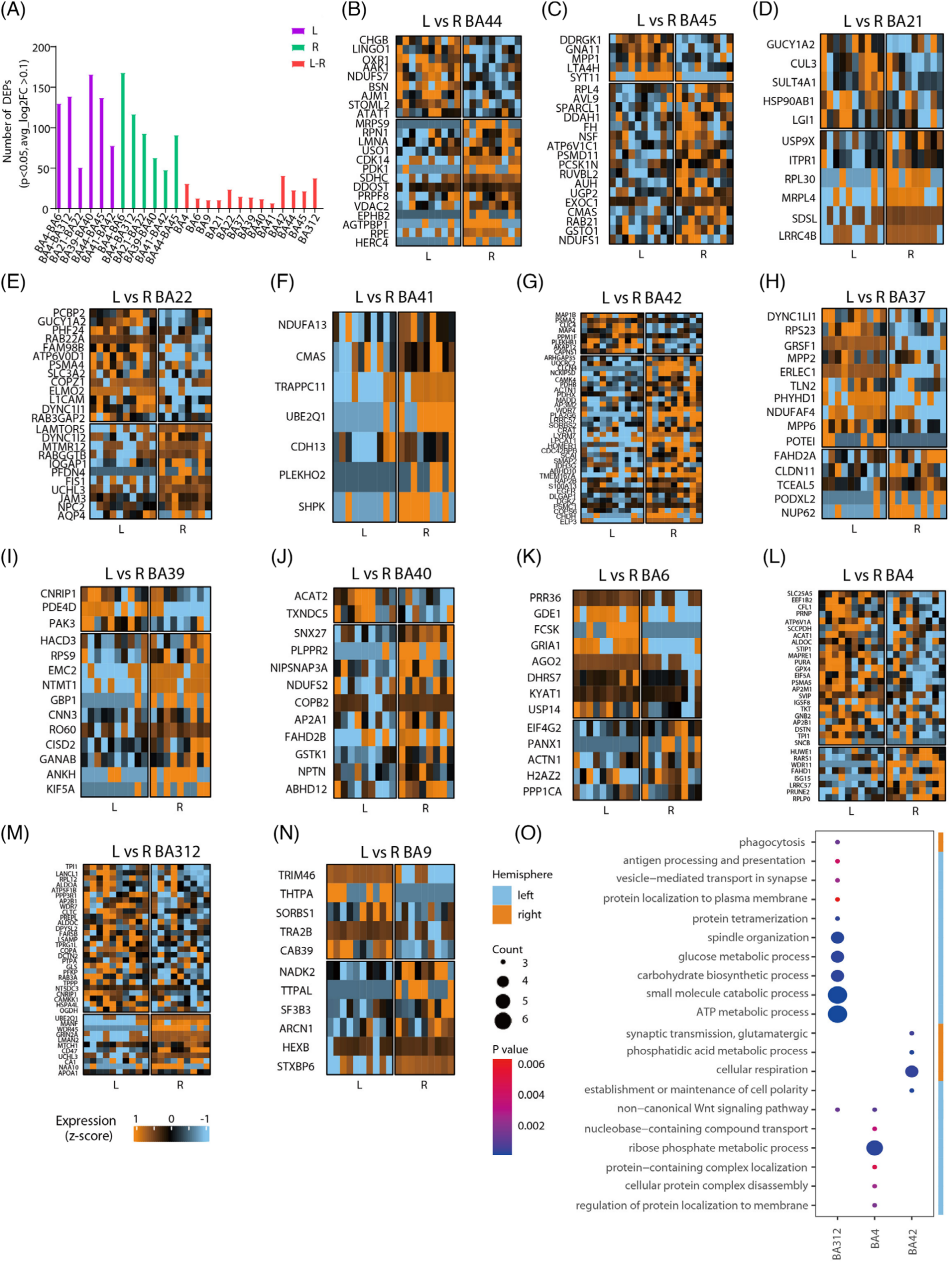
Differential protein expression patterns and enriched pathways between the left‒right paired 13 Brodmann areas (BAs). (A) Differentially expressed protein (DEP) number of BA pairs in the left, right and left‒right hemispheres. DEP was set as avg_log_2_FC > .1 and *p* < .05. (B‒N) Heatmaps of selected DEPs of each left‒right pair of the 13 BAs. Expression was shown as *z*‐score. DEPs were set with *p* < .05 and avg_log_2_FC > .1. For those DEPs with avg_log_2_FC ≥ 5, they were indicated in the heatmaps. (O) In the differential expression analysis of each left‒right BA pair, only BA312, BA4 and BA42 showed gene ontology‐biological process (GO‐BP) functional enrichment, as illustrated in the bubble plot. FDR < .05 and gene count > 2.

Left‒right homologous BA pairs exhibited few DEPs (<50; Figure 4A), suggesting that subtle molecular asymmetries may support functional lateralisation. In Broca's area, left BA44/45 DEPs (e.g., LINGO1,^53^ SYT11) are linked to language and synaptic function, while right BA44/45 DEPs (e.g., EPHB2,^54^ PSMD11^46^) are more related to emotion and memory (Figure 4B,C and Table S11, avg_log_2_FC ≥ 5 were shown), consistent with their functional specification. Similarly, in Wernicke's area, left BA21/22 DEPs (e.g., LGI1,^55^ L1CAM^56^) are involved in speech development, whereas right BA21/22 DEPs (e.g., USP9X,^57^ AQP4^58^, ^59^) are linked to verbal fluency and memory (Figure 4D,E), supporting bilateral involvement in semantic processing. In the auditory cortex, left and right BA41 showed near‐identical profiles except for TRAPPC11,^60^ while BA42 showed substantial asymmetry. Right BA42 DEPs were enriched in memory‐related (e.g., ARHGAP35,^61^ HOMER1^62^) and language‐related (e.g., CLCN4, WDR7 and PLA2G6^57^, ^63^, ^64^, ^65^) proteins (Figure 4F–G), consistent with its prosody‐processing role.^54^, ^66^ Left BA37, involved in visual‒semantic integration,^18^ showed no speech/memory‐related DEPs (Figure 4H). In Geschwind's territory, left BA39/40 DEPs (e.g., PDE4D) related to verbal learning,^67^ while right BA39/40 DEPs (e.g., SNX27) were associated with memory^68^ (Figure 4I,J), mirroring their functional specialisation in language processing and spatial attention.^19^

Motor‐related regions (BA6, BA4, BA3/1/2) showed the most pronounced asymmetry. Left‐sided DEPs (e.g., GRIA1, PRNP, DPYSL2) were strongly associated with speech and motor development,^55^, ^61^, ^62^, ^69^ consistent with the fact that speech motor plans are primarily controlled by the left vPMC and vSMC area. Besides, many of those DEPs are metabolic enzymes, supporting the enrichment of metabolic processes in left BA312 and BA4 (Figure 4K‒M,O and Table S12). At last, BA9, important for Chinese reading,^70^ showed minimal lateralised differences, and its top DEPs were not linked to speech/memory (Figure 4N).

Together, BA4 and BA312 within the vSMC area demonstrated the strongest molecular asymmetry, with functional enrichment suggesting a metabolic and speech motor specialisation in the left hemisphere.

### 2.6 Protein expression pattern and E/I balance underlying brain connectivity

**FIGURE 5:**
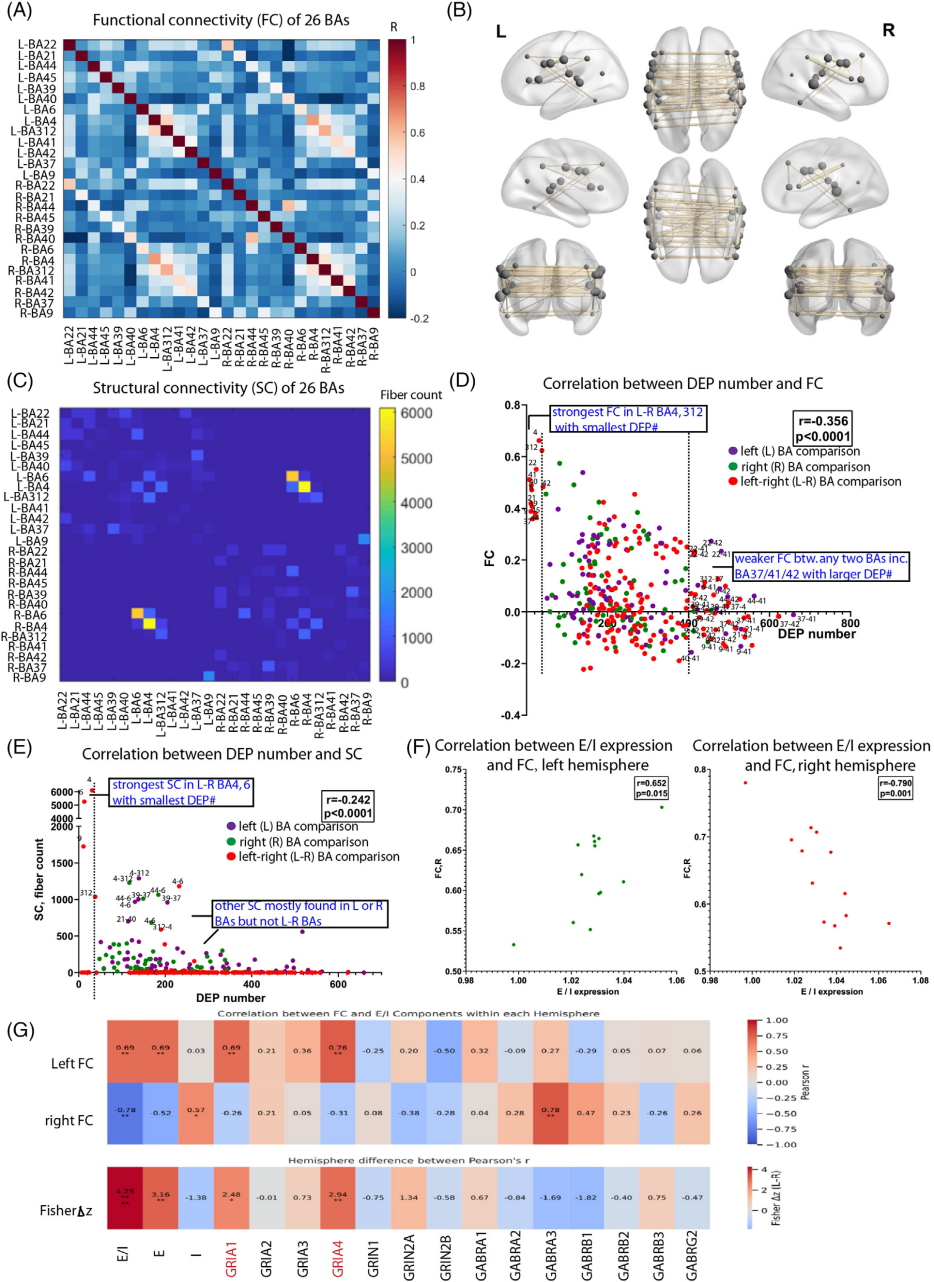
Protein expression difference between any two Brodmann areas (BAs) among the 26 BAs was negatively correlated with both functional and structural connectivity. (A) Heatmap of Pearson correlation coefficient of functional connectivity of 13 BAs in the left hemisphere and their homologous BAs in the right hemisphere. *r*: Pearson correlation coefficient. (B) Visualisation of functional connectivity of 26 BAs in the left and right hemispheres. The thickness of the lines represents the strength of functional connectivity in two BA regions. The node size represents the average strength of the functional connectivity between a given BA with all other BAs. Edges with functional connectivity value above .2 are visualised. (C) Heatmap of fibre count of structural connectivity of 13 BAs in the left hemisphere and their homologous BAs in the right hemisphere. (D) Differentially expressed protein (DEP) number of any two BA comparisons in the left (purple), right (green) or left‒right (red) hemisphere(s) was significantly negative‐correlated with functional connectivity. *r* = ‒.356, *p* < .0001. (E) DEP number of any two BA comparisons was significantly negative‐correlated with structural connectivity. *r* = ‒.242, *p* < .0001. (F) Excitatory/inhibitory (E/I) expression in each individual BA is significantly positively correlated with functional connectivity in the left hemisphere but negatively correlated with functional connectivity in the right hemisphere. (G) Top: Pearson's *r* between functional connectivity (FC) strength and E/I ratio and its excitatory (E) and inhibitory (I) components. Bottom: Fisher Δ*Z* (left–right) from Steiger tests. Asterisks mark *p* < .05 (*), *p* < .01 (**), *p* < .001 (***) and *p* < .0001 (****). The largest asymmetry is driven by the excitatory gene expression, especially GRIA1 and GRIA4. (D and E) DEP was set as *p* < .05 and avg_log_2_FC > .1.

To assess how protein profiles relate to inter‐regional connectivity, we mapped the 26 language‐related BAs with approximately 5 mm radius onto 32k_fs_LR space and constructed the group‐level resting‐state FC across 90 HCP subjects.^33^ Strikingly, each BA showed its strongest FC with its contralateral homologue rather than with any other region (Figure 5A). Visualisation highlighted these bilateral connections as the most robust, with node size reflecting overall FC strength (Figure 5B). SC by fibre count, derived from deterministic tractography, similarly showed that left‒right BA6 and BA4 pairs had the highest fibre counts, indicating strong anatomical coupling (Figure 5C).

As an alternative SC measurement, we calculated streamline density by dividing the number of streamlines between each BA pair by their average surface area. This analysis across all region pairs and participants revealed a pattern highly consistent with that of streamline count (Figure S7; *r* = .98, *p* < .0001).

We next examined the correlation between DEP number and FC across all BA pairs (*n* = 325). FC strength was negatively correlated with DEP number (*r* = ‒.356, *p* < .0001; Figure 5D and Table S13), indicating that BAs with more similar protein expression tend to exhibit stronger functional connections. Notably, the strongest FC (>.6) was observed between left‒right BA4 or BA312 with almost smallest DEP number (<40) (Figure 5D). In contrast, BA37 and pAC (BA41/42) consistently exhibited the highest protein expression divergence (>400 DEPs) and weakest FC (<.2), suggesting they are more functionally independent (Figure 5D). Together, these findings suggest that proteomic similarity underlies FC in language‐related brain regions.

Similarly, SC strength is negatively correlated with DEP number (*r* = ‒.242, *p* < .0001; Figure 5E). Strongest SC (fibre count > 5000) existed in left‒right BA4 and BA6 with few DEPs (<40). Moderate SC (500 < fibre count < 2000) was found in BA9, BA312, BA4‒BA6, BA4‒BA312, BA6‒BA44 pairs (<200 DEPs). Many left‒right pairs showed minimal SC, likely due to tractography limitations. Together, the strongest connection including both SC and FC is in left‒right BA4, and they are very similar to each other at protein expression level.

E/I receptor expression reflects brain regional heterogeneity,^71^ Due to lack of bilateral single‐cell data, we estimated E/I balance in each BA by calculating the ratio of summed excitatory AMPA (*GRIA1*, *GRIA2*, *GRIA3* and *GRIA4*) and NMDA receptors (*GRIN1*, *GRIN2A*, *GRIN2B* and *GRIN2C*), to inhibitory GABA_A_ receptors (*GABRA1*, *GABRA2*, *GABRA3*, *GABRA4*, *GABRA5*, *GABRB1*, *GABRB2*, *GABRB3*, *GABRG1*, *GABRG2* and *GABRG3*). This E/I expression ratio of 13 BAs correlated positively with FC in the left hemisphere (*r* = .652, *p* = .015) and negatively in the right (*r* = ‒.790, *p* = .001) (Figure 5F and Table S14), but showed no significant correlation with SC.

To understand this asymmetry, we compared left‒right values for FC, E/I, excitatory and inhibitory expression, confirming FC symmetry across hemispheres (Figure S8). However, FC–E correlations diverged: positive on the left and negative on the right (cocor Steiger test, *p* < .01; Figure 5G), driven mainly by GRIA1 and GRIA4. Thus, hemispheric differences in excitatory gene expression—not FC itself—explain the asymmetric FC–E/I relationships.

### 2.7 Implications of protein connectivity correlation on neural reorganisation pattern of Wernicke's glioma patients

We then included glioma patient data to test if bilateral protein expression similarity in language‐related regions supports functional compensation after damage. Proteomic analysis showed homologous areas, such as left and right Wernicke's, share similar protein profiles related to synaptic signalling and E/I balance. This molecular similarity aligns with stronger FC, suggesting that right‐hemisphere homologues can compensate when the left side is damaged. Supporting this, glioma patients with left Wernicke's tumours showed increased GMV in the right Wernicke homologue, positively correlating with language scores.

Language networks show neuroplasticity through reorganisation in lesion, perilesional, ipsilateral networks or contralesional hemispheres.^26^ Although BA4 showed the strongest bilateral connectivity with minimal protein expression differences, very few brain tumour surgeries were operated on the motor cortex (BA4) due to its pivotal role for motor control. The left‐hemispheric Wernicke's area is highly potential for neuronal reorganisation, thus we focused on left Wernicke's glioma.

Among 1124 glioma patients from two centres, 52 had Wernicke's involvement, 13 underwent linguistic tests and six (∼46%) were aphasic (Figure 1A,D and Tables 1 and S2). Compared to HCs, Wernicke patients had significantly lower linguistic scores in aphasia quotient (AQ) (*t* = 2.48, *p* = .02), repetition (*t* = 2.78, *p* = .01) and naming (*t* = 2.54, *p* = .01) (Table 1). GMV increased significantly in the right Wernicke homologue and other BAs in patients (*n* = 30 and 52 for HCs and Wernicke group, respectively, FDR < .05, Figure S9 and Table S15), indicating potential functional compensation.

**TABLE 1:** Demographic information and linguistic scores for the healthy controls (HCs) and patients with glioma involving Wernicke's area.

	HC (*n* = 30)	Patients (*n* = 52)	*χ* ^2^ or *t*	*p*‐value
Subjects for macrostructural analysis				
Age	48.30 ± 6.35	48.44 ± 12.03	.06	.95
Sex (M/F)	14/16	24/27	.001^a^	.973
Tumour volume (cm^3^)	–	140.1 ± 77.77	–	–
WHO grade	–	1/2/3/4 = 1/12/8/30	–	–

	HC (*n* = 14)	Patients (*n* = 13)	*t*	*p*‐value
Subjects with linguistic scores				
AQ	98.2 ± 2.51	89.37 ± 13.09	2.48	.02
Spontaneous speech	19.64 ± .84	17.69 ± 3.82	1.87	.074
Comprehension	224.21 ± 6.5	2133.69 ± 27.447	1.39	.176
Repetition	99.93 ± .27	89.69 ± 13.79	2.78	.01
Naming	97.14 ± 3.21	88.08 ± 112.95	2.54	.012

Abbreviation: AQ, aphasia quotient.

^a^ Pearson's chi‐square test.

**FIGURE 6:**
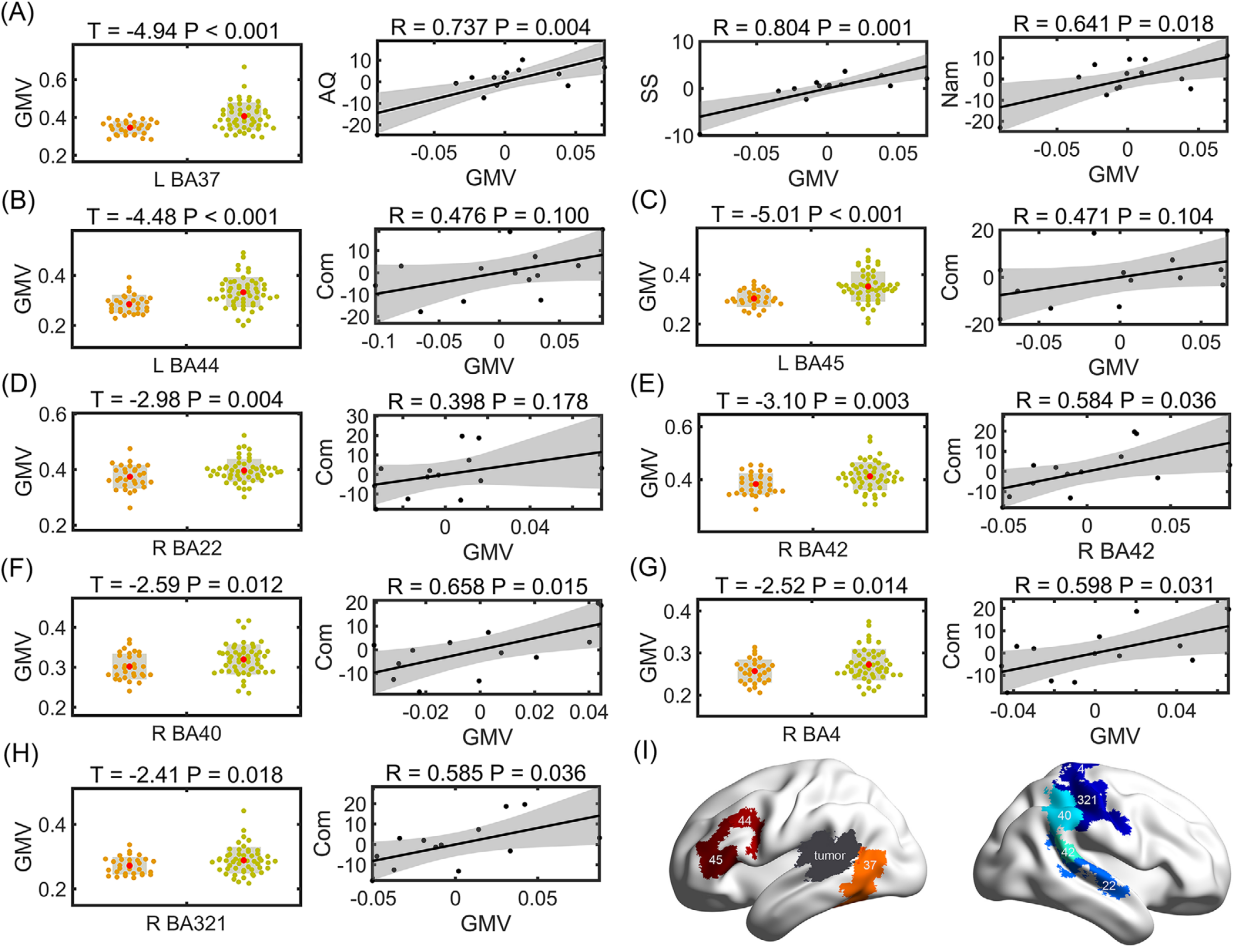
ROI‐based grey matter volume (GMV) increases in the Wernicke patients and their associations with linguistic scores. (A–H) The left panels showed ROI‐based GMV comparisons between patients (*n* = 52, chartreuse dot) and healthy control (HC) (*n* = 30, brown dot). *p* < .05, FDR correction. The right panels were scatter plots illustrating partial Pearson correlation between GMV and linguistic scores, along with corresponding linear fitted line and 95% confidence interval. (I) A summary of functional compensation in the Wernicke patients. The areas in dark grey indicated the core tumour territories in our patient group. Note that the tumour covers BA22 and BA42. Broadman areas that exhibited functional compensation for language were labelled. AQ, aphasia quotient; BA, Broadman area; Com, comprehension; FDR, false discovery rate; L, left; Nam, naming; R, right; ROI, region of interest; SS: spontaneous speech.

In the left hemisphere, only GMV in BA37 (adjacent to Wernicke's) correlated positively with linguistic scores of AQ, spontaneous speech and naming (*p* = .004, .001 and .018, respectively; Figure 6A). Other regions such as left BA44 and BA45 did not show significant correlations with linguistic scores although they had increases in GMV (Figure 6B,C). In contrast, right hemisphere homologues of Wernicke's (BA22/42) showed increased GMV and positively correlated with comprehension (Figure 6D,E). Significant correlations were also observed in right‐hemispheric homologue of Geschwind's (BA40) and vSMC (BA4, BA312) (Figure 6F‒H). This suggests right hemisphere compensatory plasticity, consistent with the observed molecular and functional similarity between bilateral language regions.

### 2.8 Protein expression and connectivity evidence of the interplay between core language network and auditory/motor system

**FIGURE 7:**
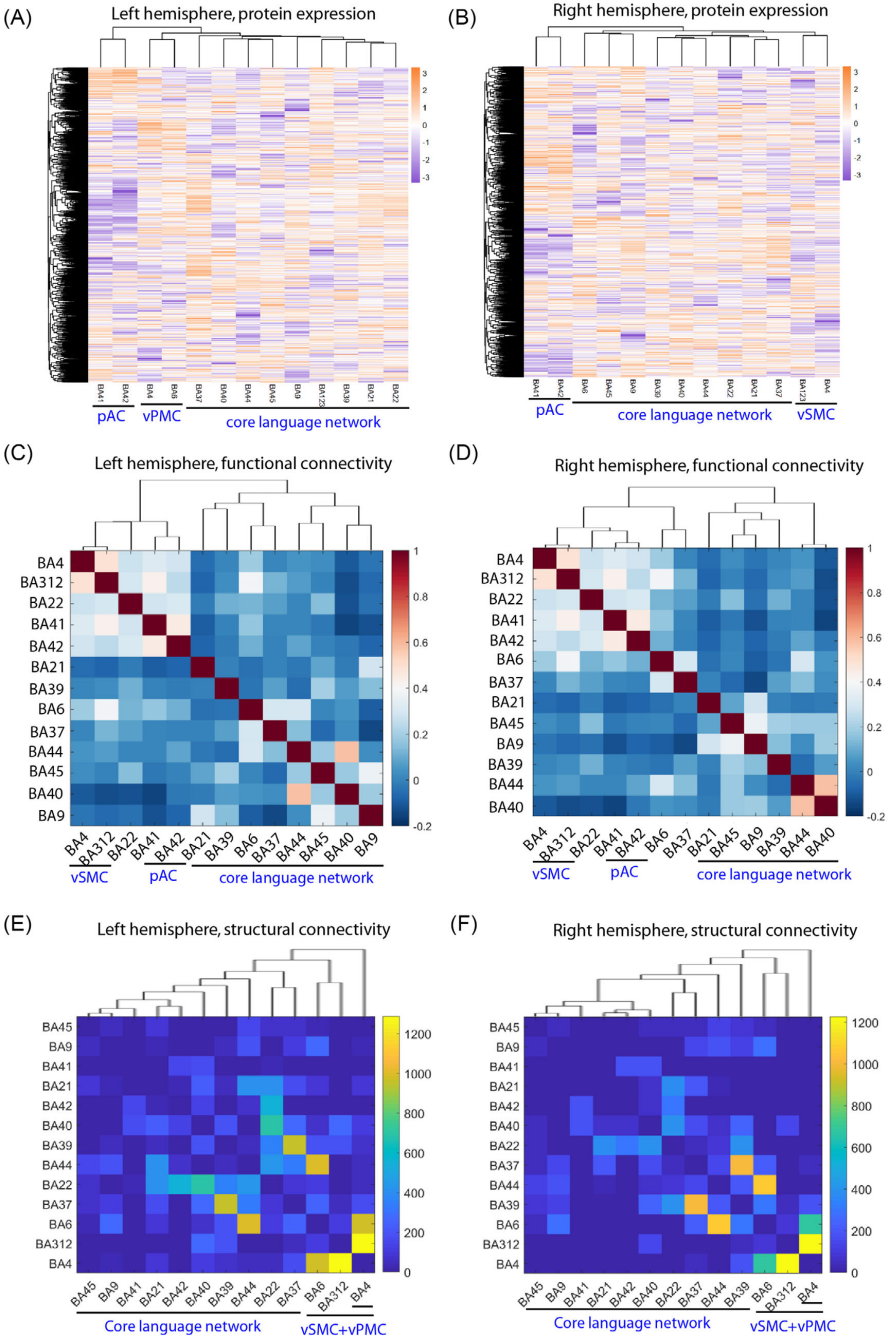
Protein expression and connectivity evidence of the independence of core language Brodmann areas (BAs) as compared to auditory and motor BAs. (A and B) Heatmap of hierarchical clustering of protein expression of 13 BAs in the left and right hemisphere, respectively. The core language network, auditory input and motor output were indicated. (C and D) Heatmap of hierarchical clustering of functional connectivity of 13 BAs in the left and right hemispheres, respectively. (E and F) Heatmap of hierarchical clustering of structural connectivity of 13 BAs in the left hemisphere (E) and right hemisphere (F).

The comprehension and production of spoken language require the interaction between the core language network, auditory input and motor output systems. The exact functional division of language and speech networks is an everlasting topic. Theoretically, a core language system of semantic and syntactic processes is distinct from a sensory‒motor interface system.^72^, ^73^ Broca's area and Wernicke's area extending to Geschwind's area are widely accepted as major nodes of the core language network.^74^, ^75^ To investigate whether the protein expression profiles of the left 13 BAs could reflect such an interplay, we performed hierarchical clustering and found that the protein expression of Broca's area and Wernicke's area extending to Geschwind's area are correlated more closely, while those of vPMC and pAC are clustered more independently (Figure 7A). Such a relationship is consistent with the interplay between core language network and auditory input and motor output.^72^, ^73^ Consistently, we also found that the hierarchical clustering pattern of FC in the left 13 BAs was remarkably similar to that of protein expression profiles, with vSMC and pAC clustering more independently (Figure 7C). In addition, we also employed a deterministic fibre tracking algorithm on multi‐shell diffusion MRI data sourced from HCP and mapped the white matter SC of the 26 BAs. Hierarchical clustering of SC in the left 13 BAs further highlighted vSMC and vPMC as the most distinct clusters (Figure 7E).

Before spoken language can be understood, the speech signal has to be pre‐processed by the auditory system in both hemispheres.^76^ This is strongly supported by our proteomic hierarchical clustering of the left and right 13 cortical BAs, respectively, showing that the pAC from both hemispheres forms a relatively independent cluster compared to the other 11 BAs (Figure 7A,B). Hierarchical clustering of functional connections also supported the independence of the pAC (Figure 7C,D). Consistently, the differential protein expression analysis also indicated that pAC is very unique from other BAs, displaying the largest number of DEPs and weakest functional connections with other BAs in both hemispheres (Figure 5D).

Together, the hierarchical analyses of protein expression and connectivity support the interplay between core language network with auditory input and motor output in both hemispheres.

## 3 DISCUSSION

Our study integrates proteomic, connectomic and clinical data to advance understanding of the molecular basis underlying language‐related brain networks. We show that brain regions with similar protein expression profiles, particularly contralateral homologues such as left‒right BA4, exhibit significantly stronger FC, supporting a hypothesis of a ‘homophilic mixing’ effect in neural circuitry organisation. Hierarchical clustering reveals that core language regions (Broca's and Wernicke's) share tightly coupled protein expression patterns, distinct from auditory and motor regions, mirroring their functional segregation. In glioma patients with Wernicke's area involvement, increased GMV in right‐hemisphere homologues correlates positively with preserved language performance, suggesting that proteomic similarity supports functional compensation and neuroplasticity.

We provide a multiregional proteomic map of 26 bilateral BAs from 10 adult brains, surpassing previous single‐brain multiregional studies (19 bilateral areas of one brain,^70^ 29 left cortical BAs of one brain^43^) and complementing receptor‐based analyses (15 neurotransmitter receptors across key language‐related regions^68^) showing hemispheric differences in E/I balance. Notably, we found that E/I ratio correlated positively with FC in the left hemisphere but negatively on the right, largely driven by AMPA receptor subunits GRIA1 and GRIA4 (Figure 5H). The hemispheric asymmetry in excitatory gene expression, rather than connectivity strength alone, underlies the asymmetric FC‒E/I relationships observed.

Wei et al.^77^ created a spatiotemporal proteomic atlas of foetal‐to‐neonatal brain development in cynomolgus monkeys, revealing stage‐specific protein dynamics linked to neurodevelopmental risk. The Misic group^1^ pioneered the biologically annotated connectome by overlaying molecular features onto connectivity patterns, showing that brain regions with similar microstructures—such as gene expression or cell‐type composition—tend to be functionally connected, even over long distances.^78^ This fits very well with our finding that contralateral homologous BAs exhibit similar protein profiles and strong FC despite their spatial separation (Figure 5). However, previous studies have not specifically explored how region‐specific proteomic patterns shape connectivity in functionally specialised systems such as the language network.

Although our proteomic profiling focused on BAs traditionally linked to language, many (e.g., BAs 6, 9, 21, 37, 39 and 40) also support motor planning, working memory, auditory and semantic processing. While not exclusively language‐specific, these regions contribute to language‐related tasks. Their shared protein expression profiles suggest a common molecular basis facilitating coordinated function during language processing. This supports models that view language as emerging from distributed networks.

We identified distinct protein expression profiles across left‐hemisphere language‐related modules—Wernicke's, Broca's, Geschwind, pAC, vPMC and vSMC. Specifically, Wernicke's area was enriched in synaptic processes, pAC in neurofilament assembly, and vPMC in metabolic pathways. These patterns align with the idea that the core language network is molecularly distinct from auditory input‐motor output system.^72^, ^73^ Hierarchical clustering of protein expression, FC and SC across the 13 ipsilateral BAs further supports this segregation. To our knowledge, this is among the first studies to provide proteomic and connective evidence linking core language network and auditory/motor system.

We next examined protein expression differences within each functional module to clarify sub‐regional specialisation. BA21 and BA22, as well as BA41 and BA42, showed minimal differential expression, suggesting functional similarity. In contrast, BA39 and BA40 exhibited clear molecular distinctions, consistent with BA39's role in visual speech and BA40's involvement in phonological and semantic processing, with BA39 enriched in metabolic pathways. Likewise, BA44 and BA45 showed distinct profiles, reflecting their roles in syntactic and lexico‐semantic processing, respectively, with BA45 enriched in myelination‐related proteins. In vPMC, BA6 (speech output) and BA4 (articulation) differed significantly, with BA6 enriched in trans‐synaptic signalling. In vSMC, BA4 (articulation) and BA312 (sensory modulation) also diverged, with BA4 enriched in RNA metabolism and BA312 in RNA splicing. These molecular distinctions align with known functional specialisations.

Although there were few DEPs between contralateral homologous BAs, BA4 and BA312 showed the largest left‒right differences, with metabolic processes enrichment consistent with known left sensorimotor cortex activity.^79^ Few studies have examined proteomic asymmetry beyond prefrontal cortex,^80^, ^81^ Our study are among one of the first multiregional proteomic comparisons of left‒right paired BAs across frontal cortex, motor cortex, somatosensory cortex, parietal cortex and temporal cortices, creating a valuable resource for brain lateralisation studies. The differential protein expression profiles between BA pairs across hemispheres would provide a very informative resource for future molecular studies (Tables S16‒S18).

Our proteomic data showed that protein expression differences are negatively correlated with both FC and SC, aligning with growing evidence that molecular similarity supports brain connectivity.^29^, ^82^, ^83^ Richiardi et al. demonstrated that brain regions with similar gene expression, particularly those related to ion channels, exhibit stronger FC in humans and mice.^29^ French et al. found that structurally connected regions often share gene expression profiles, independent of spatial distance.^82^ Recently, Hansen et al. developed a three‐dimensional atlas of neurotransmitter receptors, showing that receptor similarity mirrors SC and FC.^83^ These studies collectively suggest that connectivity is shaped by underlying molecular architecture. Our study extends this concept by providing direct proteomic evidence—particularly in language‐related regions—linking protein expression similarity to both structural and functional coupling. This adds a critical layer to understanding how the molecular landscape supports large‐scale brain network organisation.

Our findings demonstrate functional compensation in the right Wernicke's homologue, consistent with reported homotopic plasticity in tumour patients that correlates with better recovery.^84^, ^85^, ^86^ In our Wernicke glioma cohort (*n* = 13), right‐hemisphere adaptation was consistent with our observation of homotopic connectivity and protein expression patterns, with seven of 13 patients showing minimal language impairment and a significant GMV‐linguistic performance correlation, collectively supporting bilateral language network collaboration. These observations align with known mechanisms of homotopic plasticity involving shared gene expression pattern,^87^ cellular composition^88^, ^89^ and connectivity patterns.^90^, ^91^, ^92^ Furthermore, we specifically propose that matched protein expression and strong functional connection may serve as its foundation, future studies involve causal or temporal data could substantiate this link. Notably, compensation was also observed in peri‐lesional and asymmetric unaffected‐hemisphere regions, supporting the complexity of functional compensation in brain injuries, although our exploratory findings require validation in larger cohorts given the limited sample size.

Our study still has some limitations. First, although we collected the proteomic data of 26 brain regions with approximately 5 mm radius from 10 donor brains, and we mapped the 26 BAs with the same radius onto 32k_fs_LR space and constructed resting‐state FC across 90 HCP subjects, the relatively small sample size may limit the generalisability of our findings. Nonetheless, our dataset remains valuable given the scarcity of multiregional human brain proteomic studies. Second, given the availability of fine‐grained cortical parcellations (e.g., Glasser atlas, 180 regions per hemisphere based on connectomic data^93^), the spatial specificity of the proteomic‒connectomic associations could be much improved in future studies. Third, the current dataset is limited to adult brains. Considering that peak asymmetries in regions associated with language development and functional lateralisation emerge between 20 and 26 weeks of gestation,^94^ the inclusion of foetal brain samples in future studies could provide insights into the developmental origins of language‐relevant molecular features. Fourth, deterministic tractography is known to underestimate white matter pathways in regions with complex fibre configurations, such as long‐range projections and crossing or bending fibres.^95^, ^96^ While probabilistic tractography methods offer greater sensitivity to such configurations, they are also associated with increased rates of false‐positive connections.^96^ Future work may benefit from the integration of advanced diffusion models, high‐resolution imaging protocols, and multimodal data to improve the biological accuracy and spatial completeness of white matter tract reconstruction.^96^, ^97^ At last, emerging datasets of single cell omics offer the opportunity to model cellular and molecular heterogeneity more precisely, which may further refine the mapping between brain proteomic architecture and FC.

Together, our study should help deepen our understanding of the protein basis of brain regionalisation, provide an important database for future language research, and show compelling evidence of the intricate interplay of protein expression, connectome and neural reorganisation.

## 4 MATERIALS AND METHODS

Please refer to Supporting Information.

## AUTHOR CONTRIBUTIONS

Jinsong Wu, Fengjiao Li and Shuolei Bu initiated the project. Zixian Wang and Lianglong Sun finished the project. Zixian Wang and Shuolei Bu conducted all the proteomic analyses. Binke Yuan supervised the neural reorganisation analyses. Lianglong Sun performed the connectome‐related analyses. Chen Zheng helped the E/I‒FC relationship analysis. Zhixin Bai helped the differential expression analysis. Guoquan Yan helped proteomic data collection and screening. Luhao Yang, Fangyuan Gong, Jiali Chen, Yien Huang and Wanjing Li helped data collection. Weiwei Xian, Kemin Zhu and Wenke Fan assisted in tissue dissection and processing. Jiaxuan Yang assisted in proteomic analyses. Shuai Wu, Linya You, Qiong Liu, Guomin Zhou, Gong‐Hong Wei and Wensheng Li provided critical comments on the manuscript. Russell G. Snell, Maurice A. Curtis and Tianye Jia provided critical comments. Jinsong Wu, Zixian Wang, Fengjiao Li, Shuolei Bu and Lianglong Sun prepared manuscript draft. Linya You, Jinsong Wu, Weijia Zhang, Yong He, and Binke Yuan supervised the project, prepared and finalised the manuscript.

## CONFLICT OF INTEREST STATEMENT

The authors declare that they have no known competing financial interests.

## ETHICS STATEMENT

Human postmortem brain tissue from 10 donors were obtained from Red Cross body donation site at Fudan University under ethic approval 2019C025. The study was approved by the Ethics Review Committee of Basic Medical Sciences, Fudan University. The animal experiments were conducted in accordance with the guidelines set by the Institutional Animal Care and Utilisation Committee of Basic Medical Sciences, Fudan University.

## Supporting information



Supporting information

Supporting information

## Data Availability

The main data of this project are available within the article and as Supporting Information (figures and tables). The code supporting the study will be made available upon reasonable request. The MS proteomic data have been deposited to the Proteome Xchange Consortium via the iProX partner repository and are publicly accessible at http://proteomecentral.proteomexchange.org.
