# Supplementary material for: Molecular architecture of language‐related cortical areas revealed by integrative proteomic and connectome analyses
Source: Clin Transl Med. 2025 Aug 27;15(9):e70449. doi: 10.1002/ctm2.70449 (PMC12390769; doi:10.1002/ctm2.70449)
Supplement: Supplementary file 1 — Supporting information [file CTM2-15-e70449-s001.docx]

**Molecular architecture of language-related cortical areas revealed by integrative proteomic and connectome analyses**

Jinsong Wu^1#^*, Zixian Wang^2#^, Fengjiao Li^3#^, Shuolei Bu^4#^, Lianglong Sun^5,6,7#^, Chen Zheng^8^, Limiao Liang^2^, Zhixin Bai^3^, Luhao Yang^1^, Fangyuan Gong^1^, Jiali Chen^9^, Yien Huang^9^, Wanjing Li^9^, Guoquan Yan^4^, Weiwei Xian^3^, Jiaxuan Yang^10^, Shuai Wu^1^, Kemin Zhu^3^, Wenke Fan^3^, Qiong Liu^3^, Guomin Zhou^3^, Gong-Hong Wei^2^, Wensheng Li^3^, Jing Yan^11^, Jingliang Cheng^11^, Russell G Snell^12^, Maurice A Curtis^13^, Tianye Jia^8,14-17^, Binke Yuan^9,18^*, Yong He^5,6,7,19^*, Weijiang Zhang^4^*, Linya You^3,20^*.

This supplementary materials contain 9 supplementary figures, and methods.


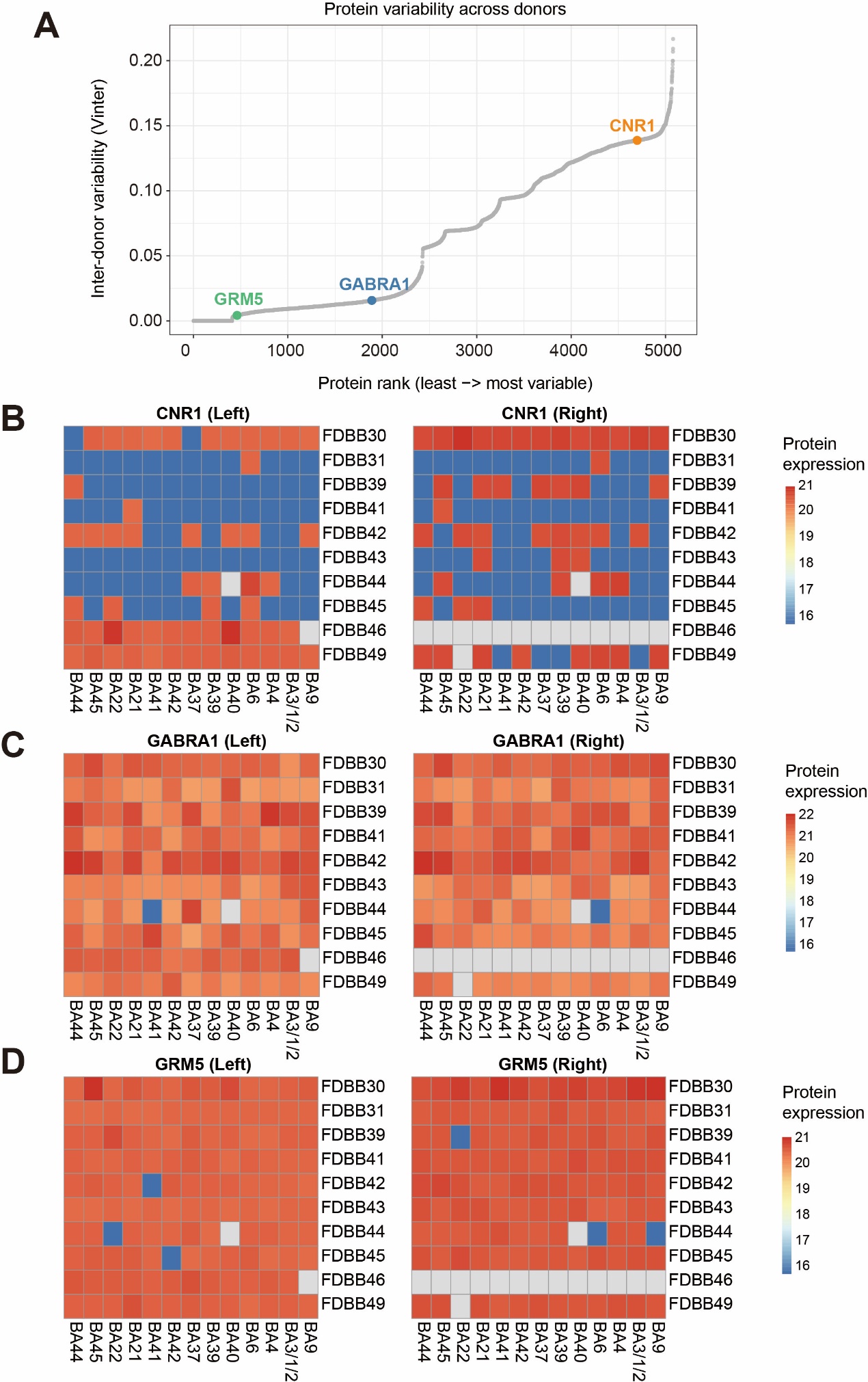


**Figure S1.** Inter-donor variability of three neurotransmitter receptors in the cortical proteome
(A) Protein-variability landscape. For each of the quantified proteins we calculated the median coefficient of variation (CV) across the 13 Brodmann areas after averaging normalised LFQ intensities within each donor. This metric is denoted V_inter​._ Proteins are ranked from the least variable (left) to the most variable (right). The cannabinoid receptor CNR1 (orange) lies near the high-variability extreme, whereas the GABA_A_​ α1 subunit GABRA1 (blue) and the metabotropic glutamate receptor GRM5 (green) fall in the low-variability regime. (B-D) Donor-by-region heat-maps for CNR1 (B), GABRA1 (C) and GRM5 (D). Normalised LFQ values are shown separately for the left and right hemispheres; rows correspond to individual donors, columns to Brodmann areas. Grey cells indicate missing measurements. CNR1 shows pronounced heterogeneity across both donors and regions, whereas GABRA1 and especially GRM5 display much more uniform abundance.


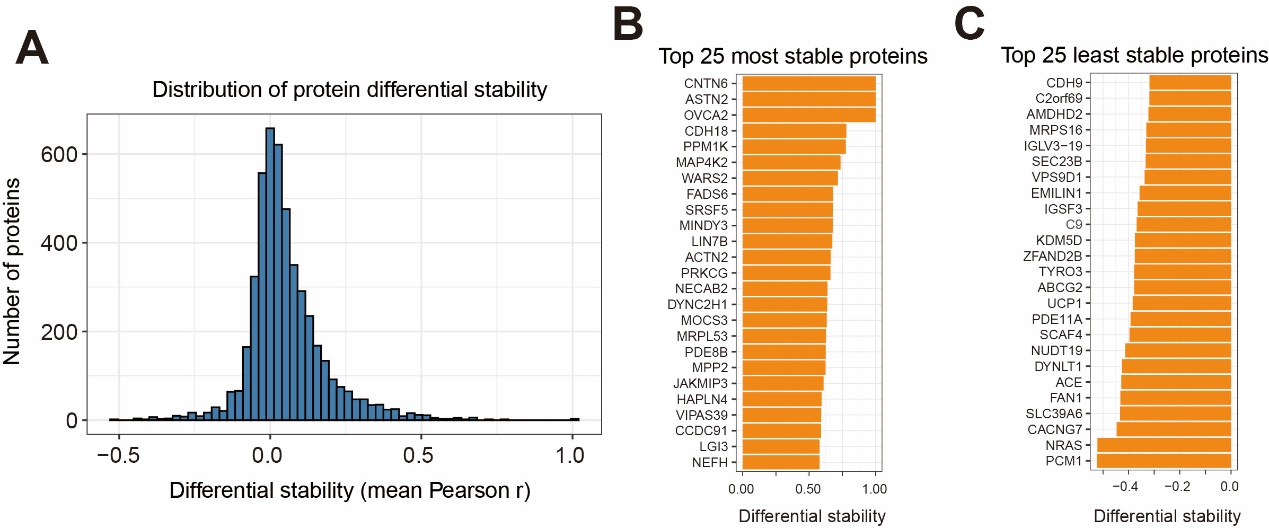


**Figure S2.** Protein differential stability across ten donor brains.
(A) Histogram showing the distribution of differential-stability scores (Δ*_S_*​) for proteins. Δ*_S_*​ is the mean Pearson correlation of each protein’s 13-region profile across the 45 donor-pair combinations. (B) Bar plot of the 25 most stable proteins (highest Δ*_S_*​ values). Bars are ordered from highest to lowest stability; Δ*_S_*​ ≥ 0.70 indicates highly reproducible regional patterns across brains. (C) Bar plot of the 25 least stable proteins (lowest Δ*_S_*​ values). Bars are ordered from most to least variable; Δ*_S_*​ ≤ -0.30 indicates pronounced inter-donor variability.


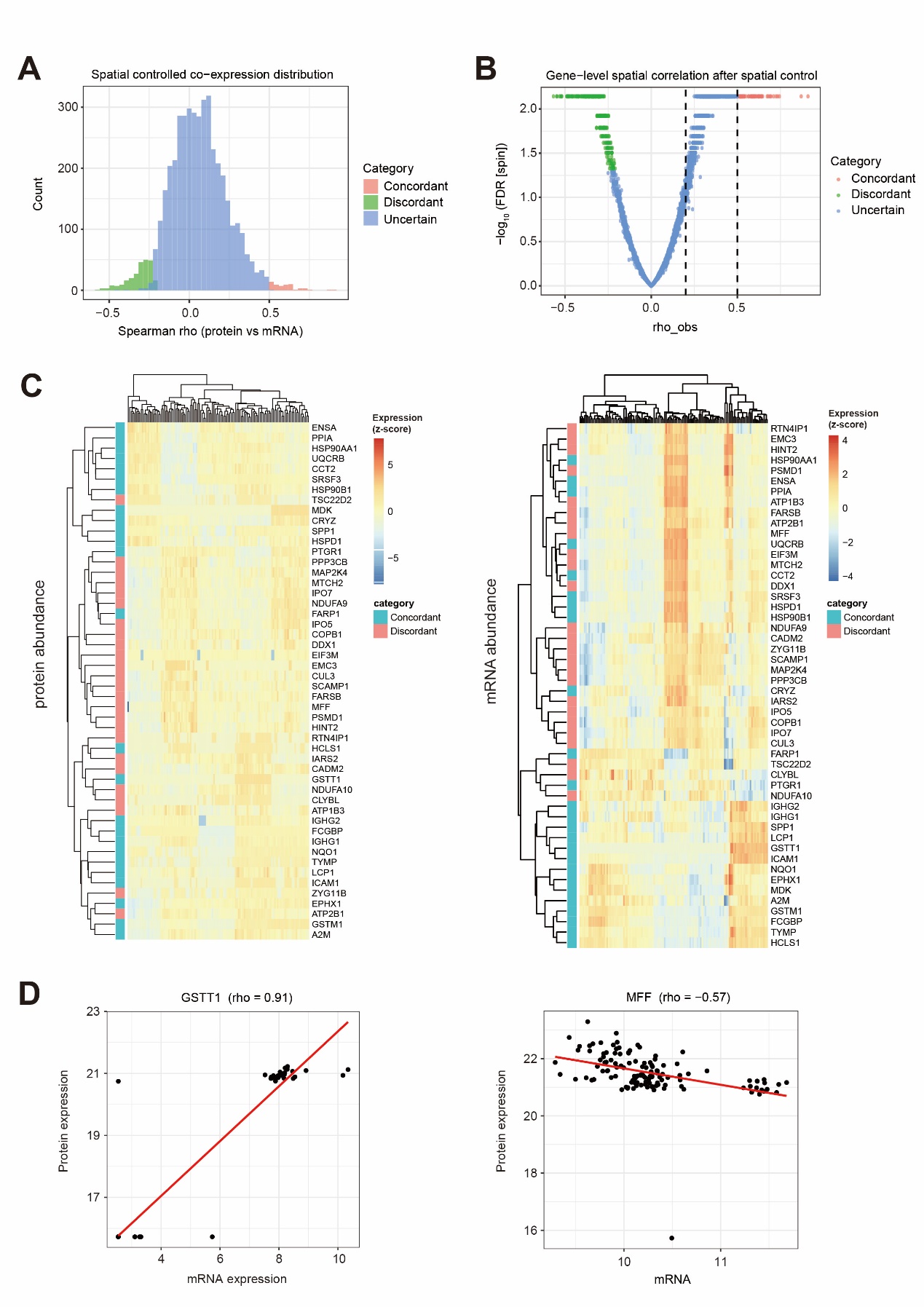


**Figure S3. Spatial concordance between mRNA and protein abundance across 13 Brodmann areas after spatial‑autocorrelation**
(A) Histogram of Spearman correlation coefficients (rho) calculated for 4,865 genes across 125 matched BA‑hemisphere samples. Bars are colored by category after spin‑test correction (FDR<0.05): positive concordance (red, n = 59), negative concordance (green, n = 255), and non‑significant genes (blue, n = 4,551). (B) Volcano‑style plot of spatial concordance (rho on the x‑axis, −log_10_FDR_spin_ on the y‑axis). Dashed vertical lines mark the classification thresholds (rho = 0.50 and −0.20).
(C) Heat‑maps of the top 25 Concordant (cyan side bar) and top 25 Discordant (salmon side bar) genes. Left panel: protein abundance; right panel: mRNA abundance. Values are Z‑scores per gene; columns are BA‑hemisphere samples clustered by Euclidean distance (Ward D^2^). (D) Representative gene scatterplots. Left, GSTT1 (rho = 0.91), an archetypal Concordant gene; right, MFF (rho = −0.57), an archetypal Discordant gene. Red lines indicate linear‑regression fits; each dot represents one BA‑hemisphere sample.


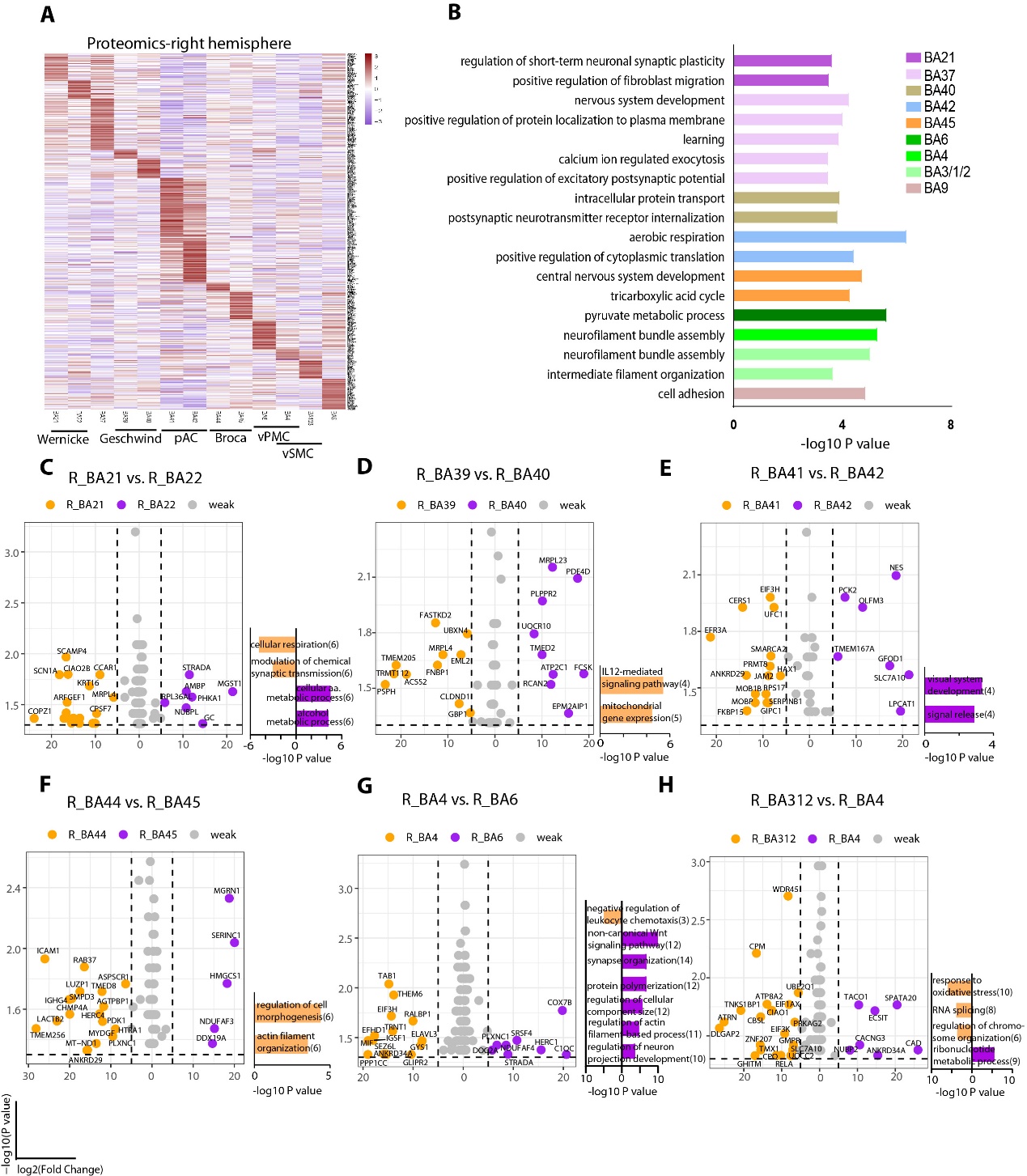


**Figure S4** Distinct protein expression patterns in the right 13 BAs

(A) Heatmap indicated the protein signatures of the right 13 BAs from 10 donors. Functional modules Wernicke, Geschwind, pAC, Broca, vPMC, and vSMC showed distinct protein expression patterns. (B) Selected GO-BP functional enrichment of DEPs of each right BA was shown. BA22, BA39, BA41, and BA44 showed no enrichment. FDR<0.05 and gene count >2. (C-H) Pair-wise differential expression and GO-BP enrichment analysis between right BA21 and BA22, BA39 and BA40, BA41 and BA42, BA44 and BA45, BA4 and BA6, BA312 and BA4, respectively. DEPs were set with p<0.05 and avg_log_2_FC >0.1. For those DEPs with avg_log_2_FC >=5, they were indicated as orange or purple dots.


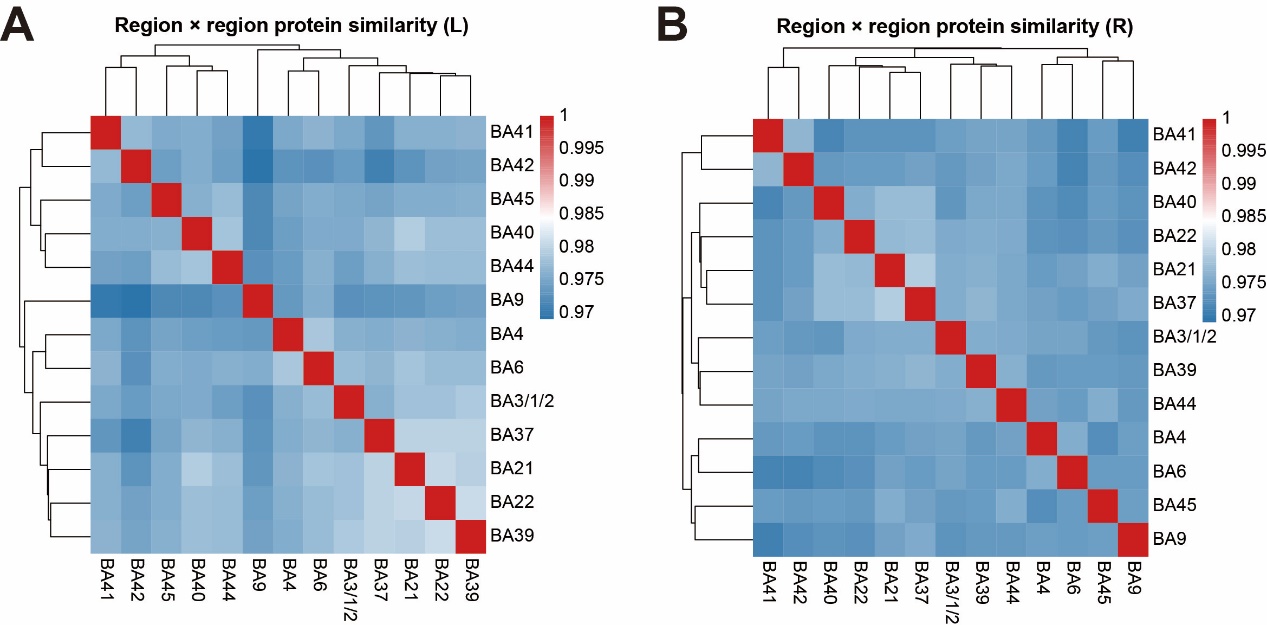


**Figure S5.** Hemisphere-specific region-by-region similarity of the cortical proteome

(A) Left hemisphere. The heat-map shows Spearman correlations between the mean protein-expression profiles of all pairs of BAs. For each BA we first averaged normalised LFQ intensities across all donors within the left hemisphere, generating one high-dimensional vector per BA. Cell colours encode the resulting correlations (common scale at right); rows and columns are clustered with Ward-D2 linkage.

(B) Right hemisphere. Matrix constructed and visualised as in (A), but using only right-hemisphere samples. Both panels share an identical colour legend so that absolute correlation values can be compared directly between hemispheres.


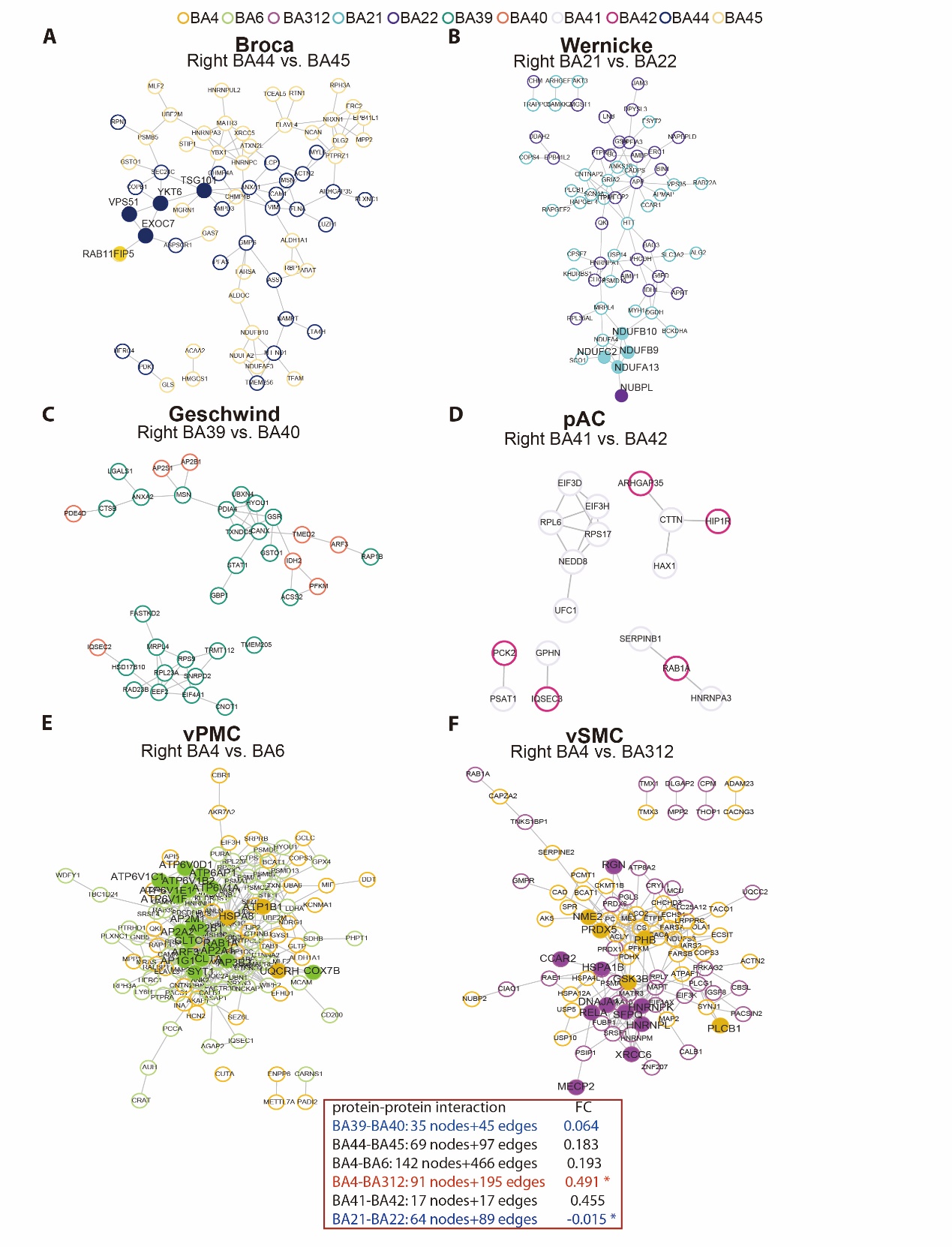


**Figure S6** STRING PPI analysis of the DEPs within each right-hemispheric homolog of language modules.

STRING PPI analysis was performed to evaluate the connectivity of DEPs between each two BAs of Broca, Wernicke, Geschwind, pAC, vPMC, and vSMC area homolog in the right hemisphere, respectively. (A) STRING PPI analysis was conducted on DEPs between BA44 and BA45 within Broca’s right homolog. The network contains 69 nodes with 97 edges. The hollow blue and yellow nodes represent DEPs from BA44 and BA45, respectively. The full colored nodes denote GO term ‘protein transport’. (B) Similar analysis was conducted on DEPs between BA21 and BA22 within the right Wernicke’s area. The network contains 64 nodes with 89 edges. The hollow cyan and royal purple nodes represent DEPs from BA21 and BA22, respectively. The full colored nodes denote ‘mitochondrial respiratory chain complex I assembly’. (C) For Geschwind’s area, the network contains 35 nodes with 45 edges. The hollow green and red nodes represent DEPs from BA39 and BA40, respectively. (D) For pAC area, the network contains 17 nodes with 17 edges. The hollow gray and violet red nodes represent DEPs from BA41 and BA42, respectively. No obvious large connected cluster was formed in the right Geschwind nor pAC area. (E) Within right vPMC area, the network of BA4 and BA6 contains 142 nodes with 466 edges. The hollow orange and light green nodes represent DEPs from BA4 and BA6, respectively. The full colored nodes form ‘vesicle-mediated transport’ and ‘hydrogen ion transmembrane transport’. (F) Within right vSMC area, BA4 and BA312 forms 91 nodes with 195 edges. The hollow orange and purple nodes represent DEPs from BA4 and BA312, respectively. The full colored nodes form ‘negative regulation of transcription’ and ‘negative regulation of apoptotic process’. The comparison of protein-protein interaction and functional connectivity strength within each module in the right hemisphere.

**
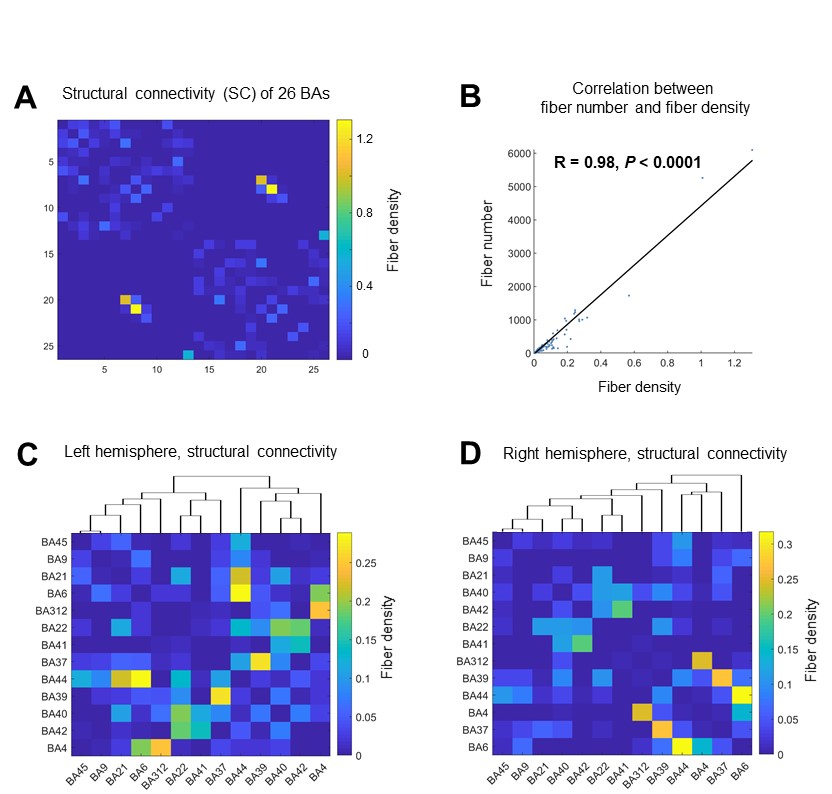
**

**Figure S7.** Validation analysis using streamline density as an alternative measure of structural connectivity
(A) Structural connectivity matrix of 26 Brodmann areas (BAs), weighted by fiber density. (B) Fiber density was highly correlated with fiber count (R = 0.98, P < 0.0001), with each dot representing a connection between a pair of brain regions. Hierarchical clustering results of the left (C) and right (D) hemisphere structural network weighted by fiber density.

**
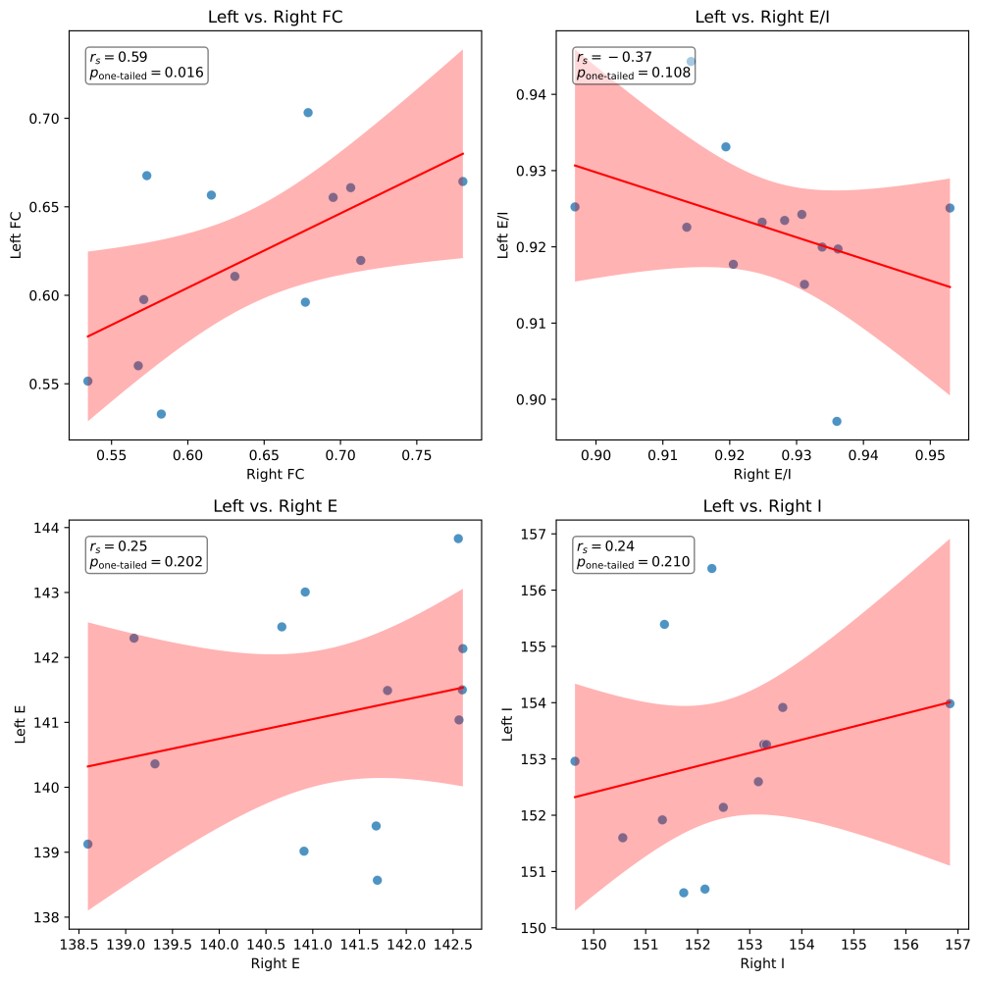
**

**Figure S8.** Scatter‐plots of left‐versus right‐hemisphere metrics across subjects

Each panel shows brain region metrics on the left hemisphere (y-axis) plotted against the corresponding right-hemisphere values (x-axis), with a red line indicating the regression fit and a shaded band denoting its 95 % confidence interval. Inset annotations report Pearson’s r and one-tailed p-values.

**
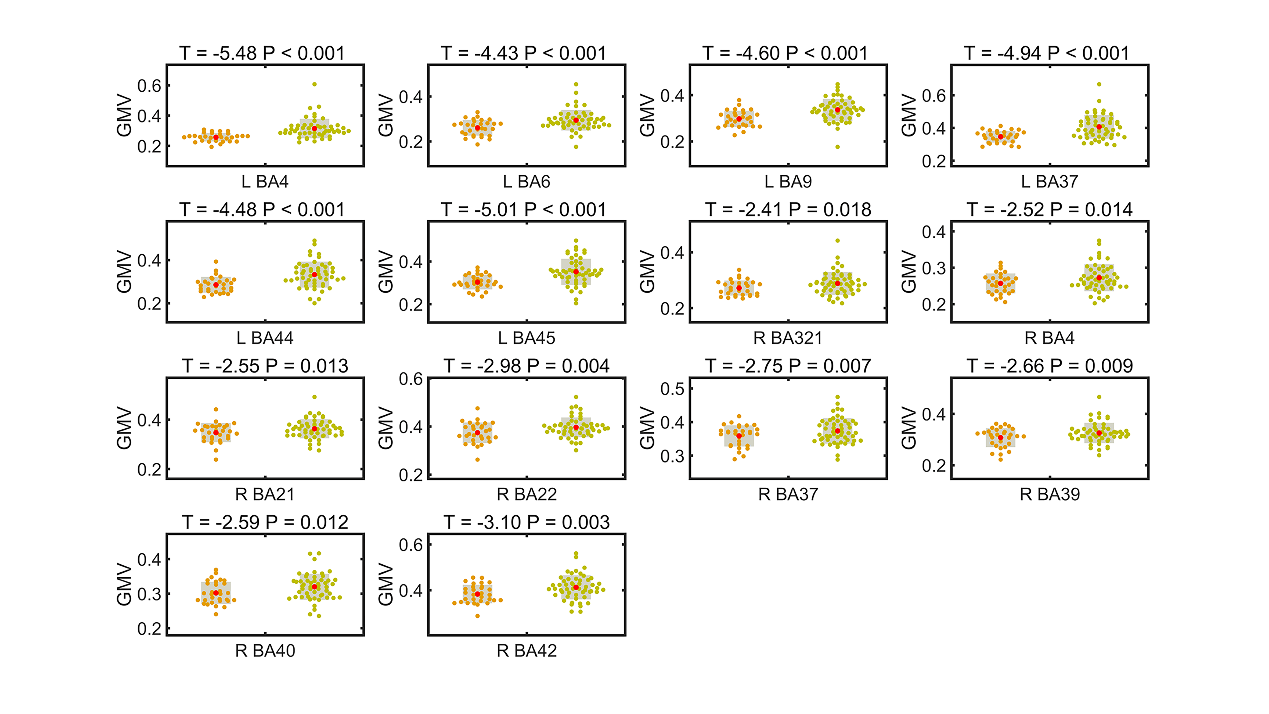
**

**Figure S9** ROI-based GMV increases in Wernicke patients (n = 52) when compared with healthy controls (n = 30). FDR correction with a corrected *P* < 0.05.

**Methods**

**Human postmortem tissue**

Human postmortem brain tissue from ten donors (Table S1) was obtained from Red Cross body donation site at Fudan University under ethic approval 2019C025. BAs 22, 21, 37, 39, 40, 41, 42, 44, 45, 9, 6, 4, 312 from both hemispheres were dissected by an experienced neuroanatomy lecturer.

**Proteome sample preparation, digestion and LC-MS/MS analysis**

Donor brains were dissected to collect specific BAs as indicated. The samples were put into cold DMEM and immediately transferred to the lab. After removing meninges and blood vessels, the samples were divided into three aliquots, one of them was used for bulk proteomics (-80℃ storage). Protein extraction and digestion were based on modifications as reported^1^. Briefly, the brain tissue was homogenized into powder by tissue homogenizer with liquid nitrogen. 100 mg of each sample was mixed with 1ml lysis buffer (8 M urea, 0.4 M ammonium bicarbonate, complete protease inhibitor), sonicated three times and cleared by centrifugation at 16,000 g, 4 ℃, 10 min. Lysates were quantified by BCA assay and adjusted to 100 μg protein in 50 μL 8 M urea, 0.4 M ammonium bicarbonate. pH was confirmed to be about 8. 5 μL 45 mM dithiothreitol was added to lysates for 30 min at 37℃, followed by an addition of 5.5 μL 100 mM iodoacetamide for 30 min in the dark at room temperature. Samples were diluted to 2 M urea with 140 μL deionized water and digested with 3 μL trypsin (at a 1 : 20 of trypin : protein ratio) for 20 hr at 37 ℃ water bath. The digestion was quenched by an addition of 2 μL 1% formic acid and the peptides were desalted using C18 Macro Spin Columns (wash 5 times by 200 μL pure acetonitrile and centrifuge at 2,300g for 1 min, followed by wash twice by 0.1 % formic acid, and add samples for centrifugation at 2,300g for 2 min). Eluted peptides were dried in a SpeedVac and stored at -20 ℃ for subsequent MS analysis.

Just before MS analysis, dried peptides were dissolved in 3.5 % formic acid and 0.1 % trifluoroacetic acid. The peptide concentrations were estimated by A280 absorbance using Thermo Nanodrop. Peptides were diluted to a final 0.04 μg/μL with 0.1 % trifluoroacetic acid, and 0.2 μg peptides were loaded for MS analysis. MS analysis was performed on a Q Exactive HF mass spectrometer (Thermo) coupled with nano ESI and nano LC (Waters). Peptides were separated using a Thermo column (2 μm, 75 μm i.d. x 50 cm) in a 0.1 % formic acid in a linear gradient of acetonitrile from 2 % to 90 % that reached 35 % acetonitrile at 175 min with a flow rate of 300 nL/min. Nano ESI voltage was set at 2.2 kV and source temperature was set at 275 ℃. MS spectrum (300-1600 m/z, target value 1x10^6^) was acquired in Orbitrap analyzer and followed by higher energy collisional dissociation-based fragmentation (normalized collision energy of 35). 20 most abundant peptide ions with a resolution of 60,000 and z>=2 were sequentially separated to a target value of 5,000 and fragmented through CID. 500 peptide exclusion list, 60 s dynamic exclusion time, 0.25 activation Q and 10 ms activation time were applied. Each sample was analyzed for 4 hrs.

Data collection and analysis were performed blind to the sample information. MS raw data were processed by MaxQuant^2^(v1.6.12.0). Spectra were searched against human UniProt (Feb 2020) using Andromeda search engine^3^. Batch effect correction and normalization were included in MaxQuant package. Quantification was performed using label-free quantification algorithm (MaxLFQ)^4^. LFQ was used to maximize the protein number identified per sample^5^ and also for comparability with other high-quality study of the mouse brain^6^.

**Downstream bioinformatic analyses**

All Statistical analysis was performed using R (version 4.0.3).

**Quality control and standardization of preprocessing data**

The quality control (QC) of proteomics data for selecting proteins was strict to remove the individual specificity. Proteins expressed in at least 5 out of 10 individuals on the left or right hemisphere for each BA are kept for downstream analysis. 3679 of 5952 proteins were reserved for differential expression analysis. Proteomics data were log2 transformed. After data preprocessing, limma^7^ removeBatchEffect function was performed to remove batch effect caused by different individuals.

**Differential stability analysis**

For each donor we first averaged left and right hemispheres so that each brain contributed one value per BA. With ten donors this yielded 45 donor-pair combinations. For each protein we then calculated the Pearson correlation between its 13-BA profile in each donor pair and took the mean of those 45 correlations as ΔS. A value close to 1 therefore denotes near-identical regional profiles in all donors, whereas values near 0 or negative indicate marked inter-individual variability.

**Spatial mRNA‑protein concordance with spin‑test**

To quantify the regional coupling between transcription and translation, we analyzed the 4,865 genes detected in both the RNA‑seq and LFQ proteomic datasets across 13 Brodmann areas. Protein intensities and TPM values were log‑transformed and donor‑wise Z‑scaled (proteomics: 10 donors; RNA‑seq: 5 donors), yielding 125 matched BA‑hemisphere samples.

For each gene we computed a Spearman correlation (rho) between its mRNA and protein levels across all samples and generated 1,000 spatial “spin”^8^,^9^ permutations to obtain an empirical *p*‑value that accounts for cortical spatial‑autocorrelation. *P*‑values were adjusted with the Benjamini–Hochberg procedure (FDR_spin_​). Genes with rho ≥ 0.50 and FDR_spin_​<0.05 were classified as Concordant; genes with rho ≤ −0.20 and FDR_spin_<0.05 were classified as Discordant; the remainder were Uncertain.

**Protein signature analysis**

For each hemisphere, each BA was compared with each of the remaining 12 BAs to obtain a panel of specific protein signature genes. For example, for BA21, there would be 12 lists of protein markers compared with the other 12 BAs, and those proteins occurred at least 5 times were selected as potential BA21 DEPs. We followed the same way to obtain DEPs for each BA, with a criterial of average log_2_ fold change > 0.1, and p value < 0.01.

**Differential expression and gene ontology (GO) analyses**

Differential protein expression between each two BAs within a functional module, or between each left and right paired BA was analyzed by a two-sided paired t-test (p<0.05), and visualized by volcano plots or heatmaps. Statistical significance was computed by student t-test and p-values were corrected using Bonferroni’s method for each pair of groups to be compared. DEPs were set with a criterial of average log_2_ fold change > 0.1, and p value < 0.05. GO-BP analyses was performed on DEPs using DAVID platform^10,11^, and the visualized by bar plots. The criterial of GO terms was p-values < 0.05, and adjust p-values < 0.05.

**STRING protein-protein interaction analysis**

The protein-protein interaction (PPI) network of DEPs between two BAs in the left and right hemispheres was evaluated using STRING database^12^ (<https://string-db.org/>). For each PPI, TSV format file containing one-way edges information was exported to be opened in Cytoscape^13^ (v3.9.1) for the next retouching.

**Hierarchical clustering analysis**

BAs with similar protein expression patterns were supposed to have similar functions. The similarity of protein expression patterns among 13 BAs were analyzed for left or right hemisphere, respectively, by hierarchical clustering^14^ using R.

**Connectome participants and data acquisition**

We used 100 unrelated subjects (54 females, 46 males, mean age = 29.1 ± 3.7 years) from the publicly available Human Connectome Project (HCP) dataset^15^ and 90 subjects passed imaging data quality control. These subjects have complete minimal preprocessed resting-state functional MRI (rs-fMRI) images, diffusion MRI images, and structural MRI images. Written informed consent was obtained from all subjects, and the scanning protocol was approved by the Institutional Review Board of Washington University in St. Louis, MO, USA (IRB #20120436).

All MRI images were acquired on a customized 3T 32-channel Siemens Skyra scanner at Washington University. T1-weighted images were acquired using a 3D-magnetization-prepared rapid acquisition with gradient echo (MPRAGE) sequence (TR = 2400 ms, TE = 2.14 ms, 256 slices, flip angle = 8°; 0.7 mm isotropic voxels, matrix = 320 × 320). T2-weighted images were acquired using a 3D T2-sampling perfection with application-optimized contrasts by using flip angle evolution (SPACE) sequence (TR = 3200 ms, TE = 565 ms). For each subject, four rs-fMRI runs were obtained by multiband gradient-echo-planar imaging acquisitions in two sessions, with two runs separately acquired per session through the phase encoding direction corresponding to left-to-right and right-to-left, respectively. The sequence parameters for each run were the same as follows: TR= 720 ms, TE = 33.1 ms, flip angle = 52°, bandwidth = 2290 Hz/pixel, field of view = 208 × 180 mm^2^, matrix = 104 × 90; 72 slices, voxel size = 2 × 2 × 2 mm^3^, multiband factor = 8, and 1200 volumes. Here, we used the rs-fMRI images from the first session. To reduce the potential influence of different phase encoding directions, we included only the left-to-right encoded run in this study.

Utilizing the Stejskal-Tanner diffusion-encoding scheme, diffusion-weighted imaging data of high spatial resolution were obtained. These data comprised isotropic measurements of 1.25 mm, encompassing 18 b0 acquisitions and 270 diffusion-encoding directions across three b-value shells (1000, 2000, and 3000 s/mm², with each shell having 90 directions). The voxel size was isotropic at 2 × 2 × 2 mm, the repetition time (TR) is 5520 ms, and the echo time (TE) is 9.58 ms.

**Imaging data quality control**

We adopted a strict quality control process to assess both structural, functional and diffusion MRI images. First, we performed the initial image quality control to exclude the low-quality images with problematic acquisitions according to the HCP’s recommended inclusion. Second, for structural images, we utilized the Euler number to assess the quality of the reconstructed cortical surface. The images with Euler number magnitude less than 1.5x the inter-quartile range in the adverse direction of the distribution (all subjects in S1200) are identified as outliers and excluded. For functional images, we excluded the subjects with large in-scanner head motion (mean FD > 0.5 mm, or the number of frames with FD over 0.5 mm > 20%). For diffusion images, we calculated the head motion indexes (i.e. mean and mean absolute deviation of the frame-to-frame displacements) of each subject during the DWI scanning sessions by using *eddy_unwarped_images.eddy_movement_rms* from minimal dMRI preprocessing pipeline. After that, we excluded subjects with one or more indexes greater than 1.5 times the inter-quartile range of the corresponding index distribution. In the last step, we visually check the quality of the preprocessed images. We excluded the structural image failure with issue segmentation, surface reconstruction, surface registration, or myelination distribution, and excluded the functional image failure with functional registration or surface mapping. Totally 10 subjects were excluded.

**Imaging data processing and functional connectivity construction**

All T-weighted and T2-weighted MRI images went through the HCP structural preprocessing pipeline^16^. The whole-brain rs-fMRI imaging data were preprocessed by the HCP minimal functional pipeline^16^, including gradient distortion correction, motion correction, echo-planar imaging distortion correction, registration to the Montreal Neurological Institute (MNI) space, and intensity normalization. Then, the volume time series were mapped to the standard CIFTI grayordinates space, and downsampled to the 32k_fs_LR mesh. To further reduce the effects of nuisance covariates, we regressed out the white matter, cerebrospinal fluid, global signals, and the 24 head motion parameters, performed temporal bandpass filtering (0.01-0.08 Hz) using SPM12 (https://www.fil.ion.ucl.ac.uk/spm/) and GRETNA^17^, and smoothed using a 6 mm full-width half-maximum (FWHM) kernel on the surface.

According to the anatomical position in postmortem tissue, we precisely located 26 BAs of both hemispheres in 32k_fs_LR HCP surface space. Specifically, take BA44 as an example, we first estimated the center of mass of the postmortem tissue in the standard 32k_fs_LR surface space and used this position as the center to define a circle with a radius of 5mm. To ensure that the partition did not exceed the prior boundary, the final BA44 region was determined by the intersection of the vertices within this circle and the vertices encompassed by the entire BA44 partition of Brodmann Atlas.

For each subject, we obtained the mean time series of vertices in each BA region and calculated Pearson’s correlation coefficient of the time series between any pair of regions to construct functional connectivity. The group-average functional connectivity between all BAs was constructed across subjects.

**Structural connectivity construction**

The diffusion imaging data were reconstructed using DSI Studio software (https://www. nitrc.org/projects/dsistudio). First, an SRC file was created from the dMRI image. Then, the spin distribution function (SDF) maps were generated using the generalized q-sampling imaging (GQI) algorithm^18^ with the diffusion sampling length ratio set to 1.25. The GQI is a model-free method to estimate the water diffusion anisotropy, which is suitable for the reconstruction of crossing fibers^19^. Deterministic fiber tracking^20^ was performed across the whole cortex in the individual native dMRI space. The process involved tracking one million fibers from a source data file processed with the GQI algorithm. An Otsu threshold of 0.6 was applied for image segmentation during tracking. The algorithm was configured to delete repeated fibers. The maximum turning angle was restricted to 45 degrees, with a step size of 0.625 mm. Fiber paths were not smoothened, and the fiber lengths were constrained between 10 mm and 250 mm. Seed points for tractography were randomly placed in the white matter mask, and fiber tracking was terminated based on the gray matter mask. We transformed the surface-based entire BA regions into each individual’s native volume space by using HCP Workbench's command label-to-volume-mapping. These atlas labels at volume space were further dilated by 2.5 mm to enter the gray matter-white matter boundary. Together, 26 BA regions represented the nodes of structural connectivity. The count of fibers between each pair of BA regions were defined as the structural connectivity.

**Neural reorganization participants and data acquisition**

**Participants**

Patients with gliomas were retrospectively enrolled from two centers, i.e., Fudan University affiliated Huashan Hospital and the First Affiliated Hospital of Zhengzhou University. Data from the two centers were analyzed together. The corresponding ethics committee approved the study. All procedures followed the Declaration of Helsinki. Written informed consent was obtained from each participant.

Inclusion criteria were as follows: (1) Pathologically confirmed glioma; (2) For patients with glioma involving the Wernicke’s area, glioma located in or partially overlapped with the left posterior superior temporal gyrus (pSTG), posterior superior temporal sulcus (pSTS), and pSMG; (3) No history of chemotherapy or radiation treatment before surgery; (4) Age between 18 and 75 years; (5) Right-handedness confirmed by the Edinburgh Handedness Inventory; (6) No symptoms of motor impairment, as indicated by a grade V on the Medical Research Council (MRC) Scale for Muscle Strength^21^; (7) Chinese Han nationality; (8) No history of brain surgery; (9) No midline shifts observed in structural images, as confirmed by the *in situ* location of midline structures of the brain (corpus callosum, septa pellucidum, third ventricle, hypothalamus, and pineal region); (10) Structural images covered the whole brain, especially the whole cerebellum; (11) Good cooperation during the linguistic/cognitive evaluations; (12) No history of other major neurological or psychiatric disorders; and (13) No history of alcohol or drug abuse.

For patients met the inclusion criteria, age- and gender-matched healthy controls were selected.

**Language assessments**

Language function was assessed in detail using the Aphasia Battery for Chinese speakers (ABC) covering spontaneous speech, comprehension, repetition, naming and aphasia quotient. The ABC is the Chinese standardized adaptation of the Western Aphasia Battery and includes subscores for spontaneous speech (S_SS_) (range, 0–20), comprehension (S_Com_) (range, 0–230), repetition (S_Rep_) (range, 0–100), and naming (S_Nam_) (range, 0–100). The Aphasia Quotient (AQ) (range, 0–100) can be calculated from these items to reflect the global severity: $\text{AQ =( }\text{S}_{\text{SS}}\text{+ }\text{S}_{\frac{\text{Com}}{\text{23}}}\text{+ 0.1*}\text{S}_{\text{Rep}}\text{+ 0.1*}\text{S}_{\text{Nam}})*2$. Participant demographics and language performance are summarized in Table 1 and Table S2.

**MRI data acquisition**

The imaging protocols were identical in the two centers. All neuroimaging data were obtained using a Siemens Magnetom Verio 3.0 T MRI scanner (Siemens Medical Solutions, Erlangen, Germany). For patients with LGGs, high-resolution T1-weighted and T2-weighted fluid-attenuated inversion recovery (T2-weighted FLAIR) images were acquired with the following parameters. T1-weighted images: axial magnetization-prepared rapid gradient-echo (MPRAGE) sequence; repetition time (TR) = 1,900 ms; echo time (TE) = 2.93 ms; flip angle (FA) = 9°; inversion time (TI) = 900 ms; field of view (FOV) = 250 mm × 219 mm; matrix size = 256 × 215; slice thickness = 1 mm; voxel size = 1 mm × 1 mm × 1 mm; slice number = 176; and scanning time = 7 min 47 s. T2-weighted FLAIR images: TR = 9,000 ms; TE = 99 ms; FA = 150°; TI = 2500 ms; FOV = 240 mm × 214 mm; matrix size = 256 × 160; slice thickness = 2 mm; voxel size = 0.9 mm × 1.3 mm × 2.0 mm; slice number = 66; and scanning time = 7 min 30 s. For patients with HGGs, T1-weighted sequence images with contrast (gadopentetate dimeglumine) were acquired with the same parameters.

**Lesion mapping**

For each patient, the tumor territory was manually drawn slice by slice on the native 3D T1-weighted images. Manual tumor drawing was performed based on the contrast-enhancing tumor areas or the FLAIR hyperintense areas (necrotic areas but not peritumoral edema were included). For 3D T1-weighted images without glioma enhancement, T2-weighted FLAIR images were used as a visual reference after being coregistered to the 3D T1-weighted images. This procedure was performed manually using RANO criteria. After manually tracing the tumor, we created a 3D T1-weighted volume that lacked the tumor area (set to 0).

**Structural data processing**

To minimize the impact of glioma on spatial normalization, for each patient, the signals within gliomas were replaced by using information from the undamaged homologous region within the contralesional hemisphere^22^. Computational Anatomy Toolbox (CAT), a powerful suite of tools for morphometric analyses (http://www.neuro.uni-jena.de/cat), was used to calculate the voxelwise gray matter volume (GMV). In brief, the T1-weighted images were segmented into gray matter, white matter, and cerebrospinal fluid individually. These segmented images were then registered into the standardized templates in the ICBM 2009c Nonlinear Asymmetric space (https://www.bic.mni.mcgill.ca/ServicesAtlases/ICBM152NLin2009). The normalized volumes of gray matter were modulated with the Jacobian determinant derived from the deformation field. Site effect were corrected using Combat (https://github.com/Jfortin1/ComBatHarmonization). The site-effect-corrected modulated volumes were spatially smoothed by convolution with a Gaussian kernel with a full width at half maximum (FWHM) of 8mm.

**Statistical analysis**

Independent two-sample t-tests were performed to compare age and linguistic scores. The Pearson Chi-square test was used to compare gender composition. ROI-based GMV comparisons between patients and HC were performed using independent two-sample t-tests, with gender, age, total intracranial volume as covariates. The t values were corrected by using false discovery rate (FDR), and the corrected p-value was set at 0.05. For brain regions with supra-threshold GMV in patients, partial Pearson correlations between mean GMVs and linguistic scores were calculated, with gender, age, tumor grade, tumor volume and TIV as covariates. ROIs within tumor extent were excluded.To accurately delineate the GMV changes in patients, voxelwise comparisons between patients and HCs were also conducted. In this analysis, intact regions within the 13 BAs were used as an inclusive mask. Voxelwise FDR-correction was applied with a corrected p-value of 0.05 and a minimum cluster size of 20 voxels.

**References**

1. Carlyle BC, Kitchen RR, Kanyo JE, et al. A multiregional proteomic survey of the postnatal human brain. *Nat Neurosci*. 2017;20(12):1787-1795.

2. Cox J, Mann M. MaxQuant enables high peptide identification rates, individualized p.p.b.-range mass accuracies and proteome-wide protein quantification. *Nat Biotechnol*. 2008;26(12):1367-1372.

3. Cox J, Neuhauser N, Michalski A, Scheltema RA, Olsen J V., Mann M. Andromeda: A peptide search engine integrated into the MaxQuant environment. *J Proteome Res*. 2011;10(4):1794-1805.

4. Cox J, Hein MY, Luber CA, Paron I, Nagaraj N, Mann M. Accurate proteome-wide label-free quantification by delayed normalization and maximal peptide ratio extraction, termed MaxLFQ. *Mol Cell Proteomics*. 2014;13(9):2513-2526.

5. Carlyle BC, Kitchen RR, Kanyo JE, et al. A multiregional proteomic survey of the postnatal human brain. *Nat Neurosci*. 2017;20(12):1787-1795.

6. Sharma K, Schmitt S, Bergner CG, et al. Cell type– and brain region–resolved mouse brain proteome. *Nat Neurosci*. 2015;18(12):1819-1831.

7. Tran HTN, Ang KS, Chevrier M, et al. A benchmark of batch-effect correction methods for single-cell RNA sequencing data. *Genome Biol*. 2020;21(1):1-32.

8. Alexander-Bloch AF, Shou H, Liu S, et al. On testing for spatial correspondence between maps of human brain structure and function. *Neuroimage*. 2018;178:540-551.

9. Markello RD, Misic B. Comparing spatial null models for brain maps. *Neuroimage*. 2021;236:118052.

10. Huang DW, Sherman BT, Lempicki RA. Systematic and integrative analysis of large gene lists using DAVID bioinformatics resources. *Nat Protoc*. 2009;4(1):44-57.

11. Sherman BT, Hao M, Qiu J, et al. DAVID: a web server for functional enrichment analysis and functional annotation of gene lists (2021 update). *Nucleic Acids Res*. 2022;50(W1):W216-W221.

12. Szklarczyk D, Kirsch R, Koutrouli M, et al. The STRING database in 2023: protein-protein association networks and functional enrichment analyses for any sequenced genome of interest. *Nucleic Acids Res*. 2023;51(D1):D638-D646.

13. Shannon P, Markiel A, Ozier O, et al. Cytoscape: a software environment for integrated models of biomolecular interaction networks. *Genome Res*. 2003;13(11):2498-2504.

14. Open source clustering software. - PubMed - NCBI. https://www.ncbi.nlm.nih.gov/pubmed/?term=Bioinformatics%2C+20(9)%3A+1453-1454. Accessed May 13, 2020.

15. Van Essen DC, Smith SM, Barch DM, Behrens TEJ, Yacoub E, Ugurbil K. The WU-Minn Human Connectome Project: an overview. *Neuroimage*. 2013;80:62-79.

16. Glasser MF, Sotiropoulos SN, Wilson JA, et al. The minimal preprocessing pipelines for the Human Connectome Project. *Neuroimage*. 2013;80:105-124.

17. Wang J, Wang X, Xia M, Liao X, Evans A, He Y. GRETNA: a graph theoretical network analysis toolbox for imaging connectomics. *Front Hum Neurosci*. 2015;9:386.

18. Yeh F-C, Wedeen VJ, Tseng W-YI. Generalized q-sampling imaging. *IEEE Trans Med Imaging*. 2010;29(9):1626-1635.

19. Jin Z, Bao Y, Wang Y, et al. Differences between generalized Q-sampling imaging and diffusion tensor imaging in visualization of crossing neural fibers in the brain. *Surg Radiol Anat*. 2019;41(9):1019-1028.

20. Yeh F-C, Verstynen TD, Wang Y, Fernández-Miranda JC, Tseng W-YI. Deterministic diffusion fiber tracking improved by quantitative anisotropy. *PLoS One*. 2013;8(11):e80713.

21. Paternostro-Sluga T, Grim-Stieger M, Posch M, et al. Reliability and validity of the Medical Research Council (MRC) scale and a modified scale for testing muscle strength in patients with radial palsy. *J Rehabil Med*. 2008;40(8):665-671.

22. Nachev P, Coulthard E, Jäger HR, Kennard C, Husain M. Enantiomorphic normalization of focally lesioned brains. *Neuroimage*. 2008;39(3):1215-1226.
